# Aqueous Solution Behavior of Poly(styrene-*alt*-maleic acid)‑*b*‑poly(*N*‑acryloylmorpholine) Double Hydrophilic Block Copolymers in the Absence and Presence of Divalent Cations and Phospholipids

**DOI:** 10.1021/acs.macromol.5c01099

**Published:** 2025-06-24

**Authors:** Lauren E. Ball, Michael-Phillip Smith, Bennie Motloung, Rueben Pfukwa, Bert Klumperman

**Affiliations:** Department of Chemistry and Polymer Science, University of Stellenbosch, Matieland 7602, South Africa

## Abstract

The extraction of membrane proteins (MPs) from their native lipid environment into nanosized poly­(styrene-*co*-maleic acid) (PSMA)-lipid particles (SMALPs), allows for retention of MP structure and functionality. Furthermore, numerous proteins require metal ions (such as Mg^2+^ and Ca^2+^) to maintain their activity. Generally, SMALPs are destabilized at relatively low concentrations of divalent cation as chelation to PSMA carboxylate functional groups decreases electrostatic repulsion between SMALPs and ultimately facilitates precipitation of the copolymer and the contents of the SMALP. Double hydrophilic block copolymers (DHBCs), in which one block comprises PSMA or one of its analogues, form colloidally stable hybrid polyionic complexes (HPICs) upon exposure to M^2+^, thereby overcoming the divalent cation sensitivity of traditional PSMA macromolecular detergents. Therefore, poly­(styrene-*alt*-maleic acid)-*block*-poly­(*N*-acryloylmorpholine) (PSMA-*b*-PNAM) and its analogue poly­(4-*tert*-butylstyrene-*alt*-maleic acid)-*block*-poly­(*N*-acryloylmorpholine) (P*t*BuSMA-*b*-PNAM) were synthesized via reversible addition–fragmentation chain transfer (RAFT)-mediated polymerization and the aggregation behavior of PSMA-*b*-PNAM/M^2+^ and P*t*BuSMA-*b*-PNAM/M^2+^ complexes investigated. Under appropriate conditions, these complexes were amenable to self-assembly, while PSMA and P*t*BuSMA precipitated at significantly lower Mg^2+^/Ca^2+^ concentrations. Additionally, PSMA-*b*-PNAM/M^2+^ and P*t*BuSMA-*b*-PNAM/M^2+^ were efficient solubilizers of synthetic lipid vesicles, facilitating the formation of electrostatically and sterically stabilized SMALPs at unprecedented M^2+^ concentrations. This study effectively highlights the untapped potential of DHBCs as a new class of polymers for membrane protein related research endeavors.

## Graphical abstract

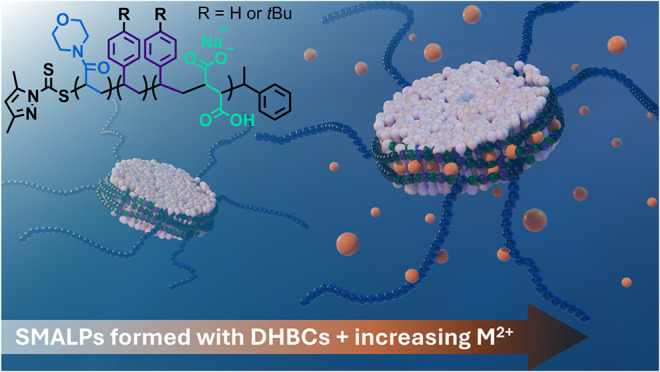

## Introduction

Poly­(styrene-*co*-maleic acid) (PSMA) is an amphiphilic copolymer with established biomedical relevance due to its application in the development of anticancer therapeutics and the structural and functional characterization of membrane proteins (MPs) which are desirable drug targets.^1–4^ Amphiphilic PSMA-type copolymers are employed as macromolecular detergents in the solubilization of MPs from the cell membrane, circumventing the use of traditional “head and tail” detergents (such as dodecyl-β-d-maltoside, DDM) and allowing for the retention of native lipids which have both functional and structural importance.^4^ Maleic acid (MAc) repeat units along the PSMA backbone are partially ionized at neutral *p*H and facilitate electrostatic repulsion, promoting an extended copolymer chain conformation. The styrene (STY) units aggregate into hydrophobic domains precluding energetically unfavorable interactions with water, promoting a collapsed coil conformation. Therefore, PSMA conformation is largely dependent on copolymer composition, comonomer distribution and charge distribution.^5^ Upon introduction to a phospholipid bilayer, PSMA associates with the membrane and undergoes conformational changes as STY pendant groups intercalate into the hydrocarbon region of the bilayer while the MAc carboxylate groups interact with the lipid headgroups and remain exposed to the aqueous medium.^6^ This causes defects in the bilayer, where an appropriate concentration of PSMA induces complete disruption of the membrane into nanodiscs, which constitute ca. 10–25 nm discoidal segments of the lipid bilayer surrounded by a PSMA belt, termed SMA-lipid particles or SMALPs. Efficient solubilization of lipid bilayers into SMALPs is only possible using PSMA copolymers with an appropriate balance of hydrophobicity and hydrophilicity. This amphiphilic balance can be tuned through variation of the STY:MAc ratio, or via the copolymerization of alternative comonomers such as 4-*tert*-butylstyrene (*t*BuSTY) or acrylic acid (AA), yielding poly­(4-*tert*-butylstyrene-*alt*-maleic acid) (P*t*BuSMA) or poly­(styrene-*co*-acrylic acid) (PSAA) respectively, which are PSMA alternatives with enhanced hydrophobicity.^7^ Other factors that affect PSMA copolymer conformation (and therefore the solubilization efficiency) are *p*H, ionic strength or concentration of divalent cations (Mg^2+^ and Ca^2+^) in the aqueous medium, which decrease the charge density along the copolymer backbone via protonation/charge screening of the carboxylate functional groups. At *p*H < 3–4 or [Mg^2+^/Ca^2+^] > ca. 5–10 mM, significant charge screening of the MAc repeat units along the PSMA or P*t*BuSMA backbone occurs, resulting in significant aggregation of copolymer chains and subsequently precipitation of the polymer from the aqueous solution.^7^ Furthermore, these conditions significantly reduce electrostatic repulsion among SMALPs, resulting in loss of colloidal stability and precipitation of the copolymer as well as the lipid/protein components of the SMALP. Therefore, the divalent cation sensitivity of PSMA-type copolymers can pose a significant disadvantage during the functional analysis of some MPs (for example, ATP-binding-cassette/ABC transporters), limiting their potential in the field of MP research.^8–10^ Zwitterionic and cationic PSMA analogues have been shown to expand the practical *p*H and Mg^2+^/Ca^2+^ concentration range of PSMA-type copolymers, but significant divalent cation tolerance was only attainable within a limited *p*H range (<3.5).^11^ This strategy required cumbersome postpolymerization modification of poly­(styrene-*co*-maleic anhydride) (PSMAnh) and can only be applied to MPs which can be efficiently solubilized and maintain their activity under acidic conditions.^11^ It was hypothesized that the utilization of a double hydrophilic block copolymer (DHBC) with a metal-interactive block such as PSMA or P*t*BuSMA, would overcome this limitation by vastly improving the Mg^2+^/Ca^2+^ tolerance of the PSMA-type copolymer, by facilitating self-assembly as opposed to precipitation of the copolymer. This method has the potential to improve the divalent cation tolerance of the copolymer, without the need for extensive postpolymerization modification or the use of limited *p*H ranges to maintain water-solubility.

The “hydrophobic effect” is the primary driving force for the self-assembly of amphiphilic block copolymers into diverse structures such as micelles and vesicles. DHBCs lack amphiphilic characteristics as both blocks are well-solvated under normal aqueous conditions. Self-assembly can be induced via application of a stimulus (*p*H, temperature, ionic strength, chelating molecules, interaction with other polymers or substrates, etc.) which increases the hydrophobicity of the stimuli responsive block, facilitating self-assembly into structures which are colloidally stabilized by the nonstimuli responsive hydrophilic block.^12,13^ DHBCs have found widespread utility in diverse applications such as drug delivery,^14^ nanoparticle synthesis and colloid stabilization,^15,16^ and the synthesis of magnetic resonance imaging contrast agents,^17–19^ but have not yet been exploited in the field of MP research to the best of our knowledge.^20^ DHBC self-assembly, mediated via interactions with metal ions/complexes, produces structures that are generally termed hybrid polyionic complexes (HPICs).^21^ A large variety of alkaline earth metals, transition metals, post-transition metals and lanthanides have been investigated for the synthesis of HPICs, where calcium and platinum have the greatest ubiquity due to their high biomedical relevance.^21^ Polymer backbones decorated with carboxylic or phosphonic acid pendant groups are most commonly employed in HPIC synthesis.^21^ For example, the self-assembly of carboxylic acid functional DHBCs such as PNAM-*b*-poly­(2-acrylamidoglycolic acid) (PNAM-*b*-PAGA), into spherical and wormlike structures via the chelation of multivalent cations such as Al^3+^, Cu^2+^, Ca^2+^ and Zn^2+^, has been achieved.^22^ Turner and co-workers previously reported the synthesis of PSMA-type DHBCs, such as poly­((4-diethylamino)-(E)-stilbene-*alt*-maleic acid)-*block*-poly­(*N*-acryloylmorpholine) (PDEAStiMA-*b*-PNAM) and demonstrated the block copolymer’s responsivity to varying *p*H and monovalent cation concentration, but did not investigate interactions between PDEAStiMA-*b*-PNAM and divalent cations.^23,24^ Poly­(*N*-acryloyl morpholine) (PNAM) is a nonimmunogenic, noncytotoxic, biocompatible, uncharged polymer, which is highly hydrophilic due to hydrogen bond formation between water molecules and the morpholine oxygen atoms and additionally can undergo facile synthesis via (controlled) radical polymerization techniques, making it a desirable candidate for the synthesis of PSMA-based DHBCs.^25^ In addition to the two polymers described *vide supra*, PNAM-based DHBCs have been employed in the synthesis of nonviral gene delivery systems, *p*H-responsive antifouling ultrafiltration membranes, viscosity reducers for heavy oils, micropatterned surfaces for cell immobilization and/or alignment as well as thermo- and photoresponsive hydrogels as carriers for synergistic anticancer therapeutics.^26–31^

In this study we investigated the synthesis of novel DHBCs, PSMA-*b*-PNAM and P*t*BuSMA-*b*-PNAM, via reversible addition–fragmentation chain transfer (RAFT)-mediated polymerization and characterized their solution properties in aqueous media with variable *p*H, [Mg^2+^/Ca^2+^] and polymer concentration (Scheme 1). The solubilization of model synthetic lipid vesicles (constituting 1,2-dimyristoyl-*sn*-glycero-3-phosphocholine, DMPC) at varying divalent cation concentration was investigated, to evaluate the applicability of DHBCs in MP research.

**Scheme 1:**
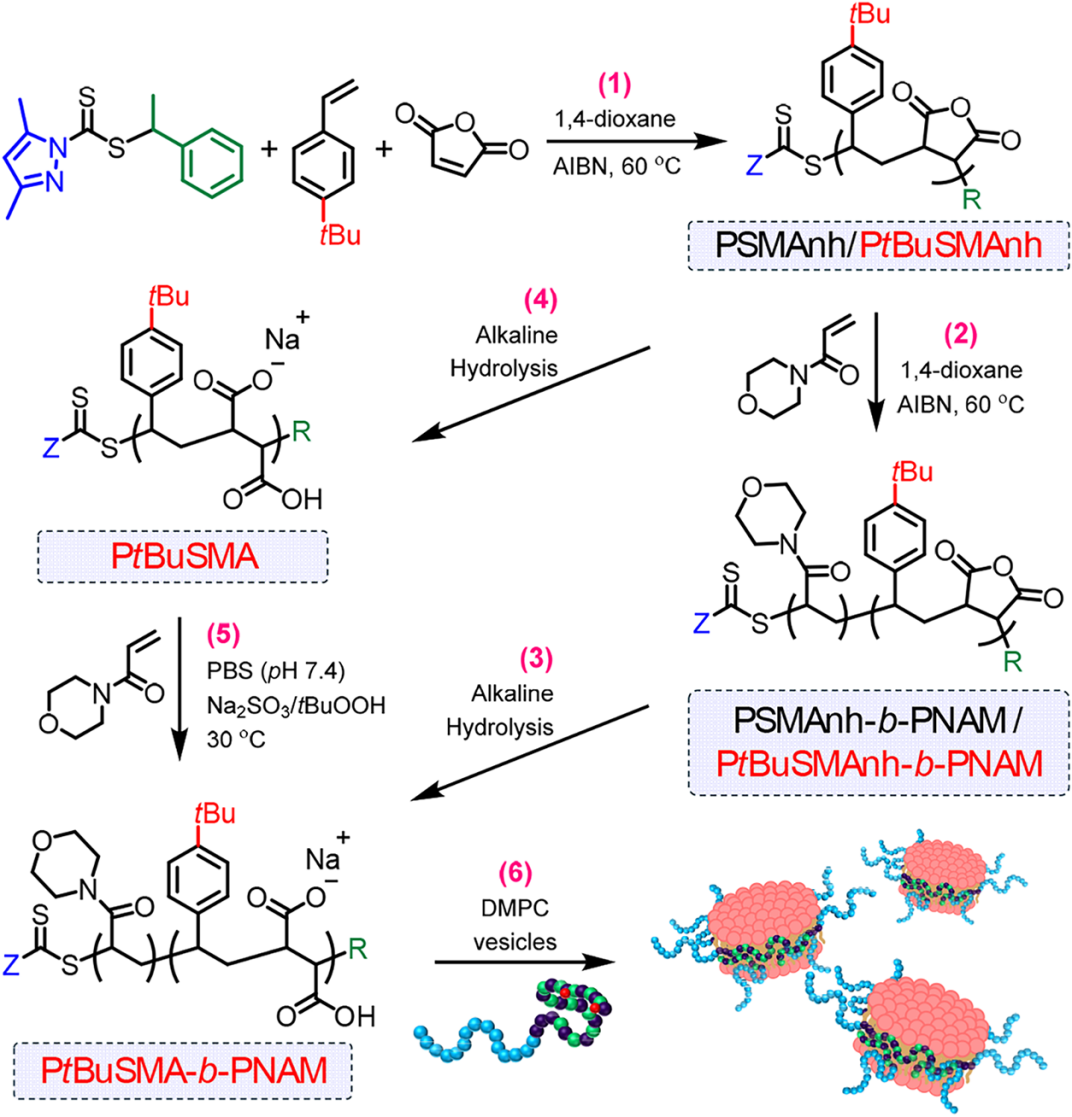
(1) RAFT-Mediated Copolymerization of MAnh and STY (or *t*BuSTY with Inclusion of Red *t*Bu Group) Yielding PSMAnh or P*t*BuSMAnh, Resp., Followed by **(2)** Chain Extension with PNAM in Organic Solvent Yielding the Amphiphilic PSMAnh-*b*-PNAM or P*t*BuSMAnh-*b*-PNAM Block Copolymers, Resp., Which **(3)** Undergo Alkaline Hydrolysis to Afford the DHBCs PSMA-*b*-PNAM or P*t*BuSMA-*b*-PNAM, Resp. **(4)** Alkaline Hydrolysis of P*t*BuSMAnh to Yield P*t*BuSMA, Which can be Chain Extended with PNAM in Aqueous Media, Affording the DHBC P*t*BuSMA-*b*-PNAM. **(6)** DHBCs are Subsequently Employed for the Solubilization of DMPC Vesicles into SMALPs at Variable Divalent Cation Concentrations

## Results and Discussion

### Synthesis of Macro-CTAs

The utility of “universal” RAFT agents for the well-controlled polymerization of various MAMs and LAMs has been demonstrated previously.^32,33^ Historically, the synthesis of well-defined PSMAnh-type copolymers and polyacrylamides such as PNAM is undertaken using trithiocarbonates and dithiobenzoates, but dithiocarbamates are relatively underutilized.^23,24,34–40^ Recently, the “universal” CTA 1-phenylethyl 3,5-dimethyl-1*H*-pyrazole-1-carbodithioate (PEPC) successfully facilitated the RAFT-mediated synthesis of well-defined PSMAnh-type copolymers.^41,42^ In the present study, the RAFT-mediated synthesis of PNAM (entry 1, Table 1) was investigated, to assess the efficiency of PEPC for the well-controlled polymerization of NAM. Quantitative monomer conversion was achieved and successful synthesis of PNAM was confirmed via ^1^H NMR spectroscopy (Figure S1). SEC analysis showed that PNAM with low dispersity (*Đ* = 1.11) could be synthesized, confirming the suitability of PEPC for the polymerization of NAM (Figure S1). Integration of signals corresponding to the R-group phenyl protons (7.12–7.44 ppm) and Z-group pyrazole proton (6.34 ppm) would suggest only 81% retention of the thiocarbonyl thio chain end was achieved, which may suggest some thermal lability of the thiocarbonyl thio moiety.^42^ Some discrepancy between *M*_n_^theo^ and *M*_n_^SEC^ was observed, likely due to the difference in hydrodynamic volume between PNAM and the PS calibration standards utilized.

**Table 1:** Monomer Conversion and Molecular Weight Data for Macro-CTAs and Corresponding Block Copolymers

entry	sample	reagent ratio^a^	α^STY^, α^MAnh^ (%)^b^	α^NAM^ (%)^b^	*M*_n_^theo^ (g/mol)^c^	*M*_n_^SEC^ (g/mol)^d^	*Đ* ^d^
1	PNAM	1:40:0.2		100	5900	4300	1.11
2	PSMAnh	1:32:25:0.1	91, 100		5700	5500	1.14
3	P*t*BuSMAnh	1:29:20:0.2	83, 100		6100	6600	1.25
4	P*t*BuSMA[Table-fn t1fn6]	1:29:20:0.2			6400	10,200	1.36
5	PSMAnh-*b*-PNAM	1:61:0.1		100	14,200	9300	1.13
6	PSMAnh-*b*-PNAM^e^	1:61:0.1		93	13,700	9400	1.18
7	PSMAnh-*b*-PNAM^e^	1:61:0.2		100	14,300	8300	1.23
8	P*t*BuSMAnh-*b*-PNAM	1:61:0.1		100	14,700	11,100	1.24
9	P*t*BuSMA-*b*-PNAM[Table-fn t1fn6]	1:60:0.2:0.2		100	14,900	18,400	1.55

a Ratio of reagents, where entry 1 employed [PEPC]:[NAM]:[AIBN], entry 2–3 employed [PEPC]:[STY/*t*BuSTY]:[MAnh]:[AIBN], entry 4 is a hydrolyzed derivative of entry 3, entries 5–8 employed [macro-CTA]:[NAM]:[AIBN] and entry 9 employed [macro-CTA]:[NAM]:[Na_2_SO_3_]:[*t*BuOOH].

b STY and MAnh conversion, respectively. Monomer conversions were determined *via* ^1^H NMR spectroscopy, using 1,3,5-trioxane as internal standard and eq S2.

c Calculated using eq S3

d Determined *via* SEC using THF (5% AcOH) as mobile phase and PS calibration standards.

e A repeat of entry 5 but conducted with kinetic sampling.

* Samples marked with an asterisk were analyzed *via* SEC using DMF (2 mM LiBr, 60 °C) as mobile phase and SMAnh calibration standards

In a recent study by Ball et al., the successful synthesis of universal PSMAnh and P*t*BuSMAnh macro-CTAs was reported, where copolymers with low *Đ* were synthesized with selective incorporation of either STY/*t*BuSTY or MAnh at the ω-chain end. Macro-CTAs with STY/*t*BuSTY as the terminal repeat unit at the ω-chain end were shown to improve the stability of the thiocarbonyl thio group compared to macro-CTAs with MAnh as the terminal repeat unit. As such, an excess of STY/*t*BuSTY was employed in the copolymerization feed and the synthesis of PSMAnh and P*t*BuSMAnh conducted using the same protocol as reported by Ball et al. (Table 1, entries 2–3). Monomer conversion was assessed *via* ^1^H NMR spectroscopy, where quantitative MAnh conversion and near quantitative STY/*t*BuSTY conversion was achieved. Due to the strongly alternating character of the PSMAnh and P*t*BuSMAnh copolymerizations, alternating copolymers with a DP of 50 and 40 repeat units respectively were synthesized, with approximately 4 STY/*t*BuSTY units incorporated at the ω-chain ends. The successful synthesis of PSMAnh and P*t*BuSMAnh was assessed *via* ^1^H NMR spectroscopy, where the incorporation of STY/*t*BuSTY at the ω-chain end was confirmed, as signals characteristic of the styrenic methine proton adjacent to the thiocarbonyl thio group were observed (Figure S2–3). Furthermore, SEC analysis showed that low *Đ* PSMAnh (*Đ* = 1.14) and P*t*BuSMAnh (*Đ* = 1.25) macro-CTAs were synthesized successfully, with good correlation between *M*_n_^theo^ and *M*_n_^SEC^ (Figure S2–3).

To prepare a water-soluble derivative of the P*t*BuSMAnh macro-CTA, the copolymer underwent alkaline hydrolysis, yielding P*t*BuSMA (Table 1, entry 4). The successful transformation of MAnh units to their corresponding MAc units was evaluated *via* ATR-FTIR spectroscopy (Figure S4). The disappearance of C=O stretching frequencies corresponding to MAnh units (1856 and 1779 cm^–1^) and the appearance of C=O stretching frequencies corresponding to MAc units (1696 and 1566 cm^–1^), as well as the appearance of a broad O–H stretch at 3099–3679 cm_,_^–1^ would suggest that hydrolysis was successful (refer to SI, Figure S6, for ^1^H NMR spectra of hydrolyzed copolymers).

### Synthesis of Block Copolymers

The polymerization of NAM was investigated in both organic and aqueous media, where the former employed PSMAnh or P*t*BuSMAnh macro-CTAs and the latter employed the P*t*BuSMA macro-CTA. An initialization period of 2 h was observed for the P*t*BuSMAnh-mediated RAFT polymerization of NAM (Table 1, entry 8), characterized by a low *k*_p_^app^ of 0.03 h^–1^ and monomer conversion of 6% (Figure 1). This period likely corresponds to the slow insertion of NAM at *t*BuSTY ω-chain ends, after which point the rate of polymerization abruptly increases (*k*_p_^app^ = 0.62 h^–1^) resulting in quantitative NAM conversion within 8 h. SEC analysis showed a generally linear evolution of *M*_n_^SEC^ and decrease in *Đ* (*Đ*_23 h_ = 1.24) with increasing monomer conversion, suggesting that the chain extension was well-controlled (Figure 1). *M*_n_^SEC^ corresponded well with *M*_n_^theo^ at low NAM conversion as the hydrodynamic volume of P*t*BuSMAnh and PS calibration standards correlate well, but an increasing discrepancy was observed at higher conversions as the PNAM content of the block copolymer increases. From approximately 6 h (α = 91%) a shoulder on the molecular weight distribution becomes apparent, corresponding to the formation of high molecular weight material. This could arise due to increased prevalence of termination events or branching side-reactions at high NAM conversion, the latter of which can result in high molecular weight shoulders with a UV response corresponding to ω-functional chain ends.^36^ Comparison of the P*t*BuSMAnh and P*t*BuSMAnh-*b*-PNAM (23 h) eluograms demonstrated a clear shift in the molecular weight distribution to lower elution volumes (Figure 2B). Furthermore, DOSY NMR spectroscopic analysis of the block copolymer indicated that protons characteristic of each block (*H*_a_ & *H*_b_, Figure 2A) had similar diffusion coefficients (*D* = 4.05 × 10^–7^ cm^2^/s), overall suggesting successful synthesis of P*t*BuSMAnh-*b*-PNAM.

**Figure 1:**
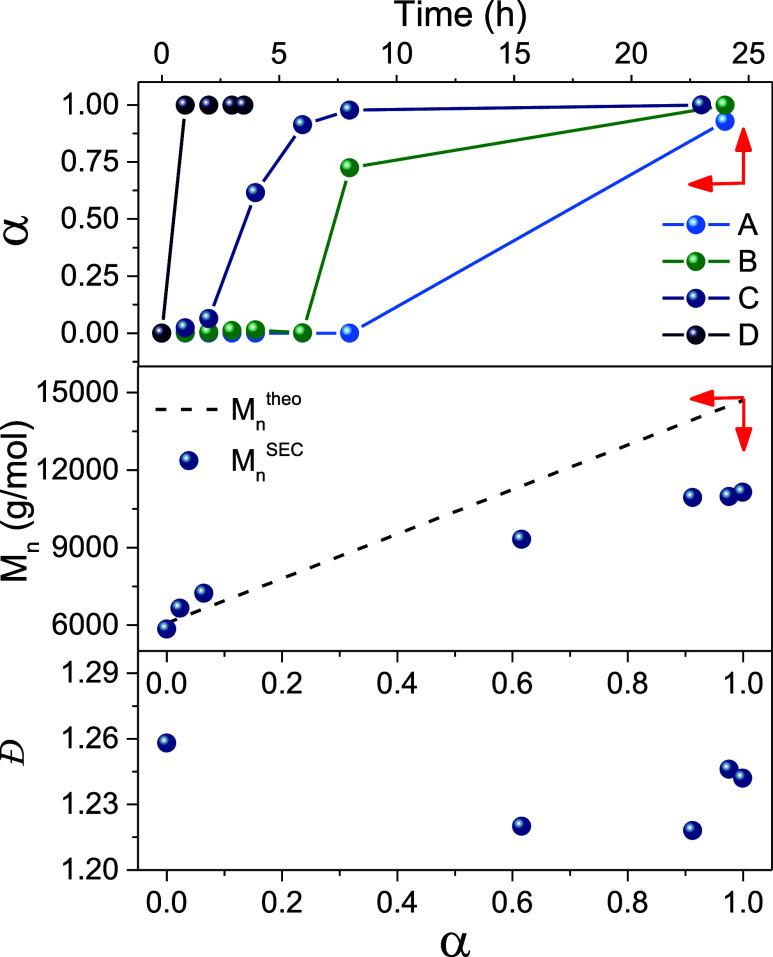
NAM conversion (α) as a function of time for BCPs synthesized in 1,4-dioxane using thermal initiation namely, (A) PSMAnh-*b*-PNAM (synthesized with 1:0.1 CTA:AIBN), (B) PSMAnh-*b*-PNAM (synthesized with 1:0.2 CTA:AIBN), (C) P*t*BuSMAnh-*b*-PNAM (synthesized with 1:0.1 RAFT:AIBN) as well as (D) P*t*BuSMA-*b*-PNAM synthesized in water using redox initiation. The evolution of *Đ*, *M*_n_^theo^ and *M*_n_^SEC^ as a function of NAM conversion (α) is also presented for P*t*BuSMAnh-*b*-PNAM.

**Figure 2:**
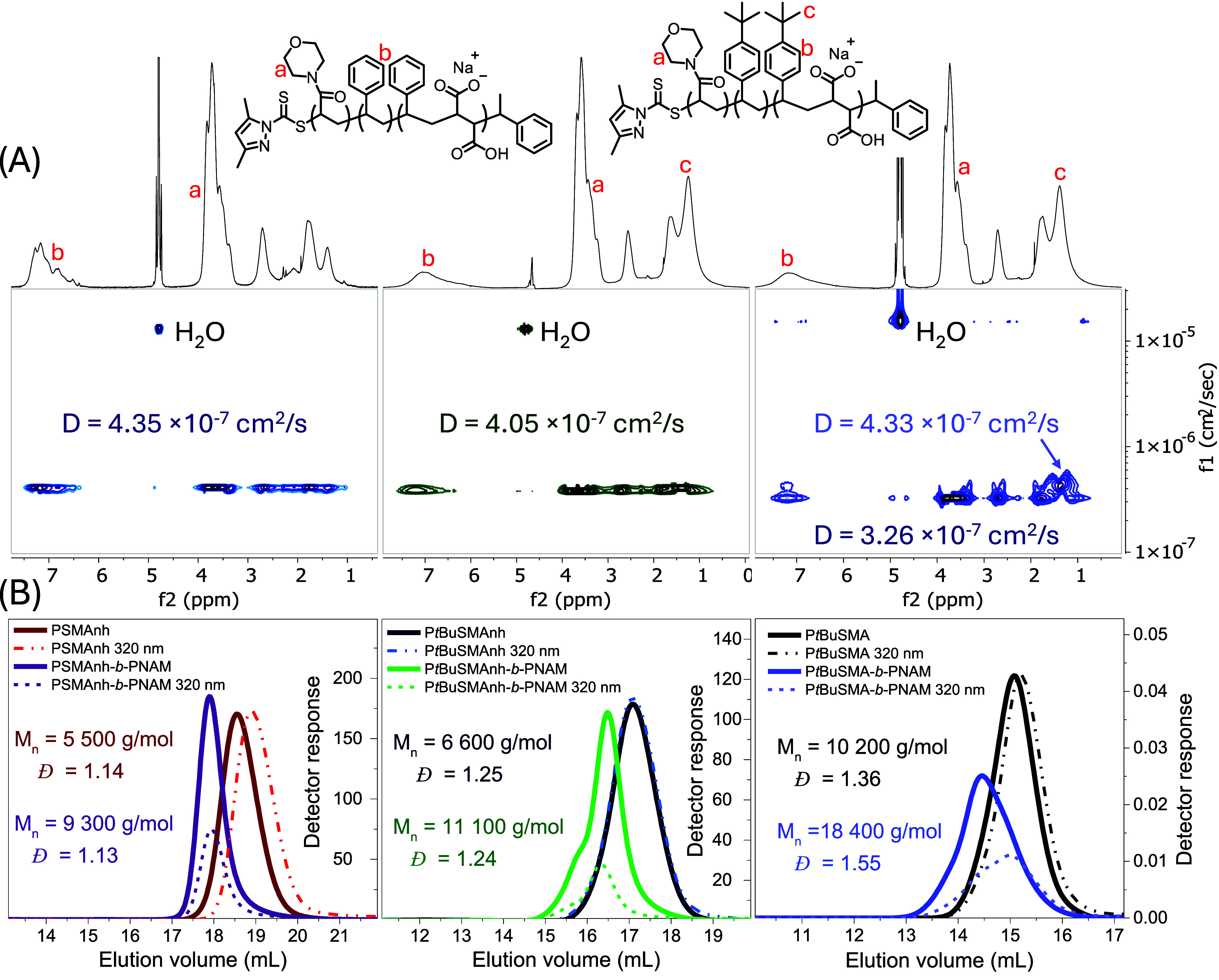
(A) DOSY NMR spectroscopic analysis (400 MHz Varian) of PSMA-*b*-PNAM (left), P*t*BuSMA-*b*-PNAM (middle) and P*t*BuSMA-*b*-PNAM synthesized in water (right) in D_2_O. All DOSY spectroscopic analyses were performed on the hydrolyzed and purified BCPs. (B) SEC analysis of PSMAnh and corresponding PSMAnh-*b*-PNAM block copolymer (left); P*t*BuSMAnh and corresponding P*t*BuSMAnh-*b*-PNAM block copolymer (middle), analyzed using THF (5% AcOH) mobile phase and PS calibration standards, as well as P*t*BuSMA and P*t*BuSMA-*b*-PNAM (right) which were acidified and analyzed using DMF (2 mM LiBr) mobile phase and PSMAnh calibration standards.

An analysis of polymerization kinetics for the PSMAnh-mediated polymerization of NAM, revealed an induction period in excess of 8 h (using a 1:0.1 CTA:AIBN ratio) or alternatively 6 h with utilization of a higher CTA:AIBN ratio (1:0.2), after which point the polymerization proceeds rapidly (Table 1, entry 6–7). This would suggest a significantly slower initialization process for the STY-based propagating radicals in combination with NAM, compared to the equivalent with *t*BuSTY-based radicals. The high molecular weight shoulder (observed for the synthesis of P*t*BuSMAnh-*b*-PNAM) became prevalent near the beginning of the polymerization where quantitative NAM conversion was achieved within 8 h. However, quantitative NAM conversion is only achieved near the end of the polymerization during the PSMAnh-*b*-PNAM synthesis, and the high molecular weight shoulder was not observed for all PSMAnh-*b*-PNAM samples (Figure 2B). SEC analysis shows a clear shift in the PSMAnh-*b*-PNAM eluogram toward comparatively lower elution volumes than the PSMAnh macro-CTA and additionally DOSY analysis of PSMAnh-*b*-PNAM indicates that PSMAnh and PNAM protons have similar diffusion coefficients (*D* = 4.35 × 10^–7^ cm^2^/s), suggesting the block copolymerization was successful.

The polymerization of NAM in aqueous media was undertaken using the water-soluble P*t*BuSMA macro-CTA and the redox initiator pair Na_2_SO_3_/*t*BuOOH, in PBS (*p*H = 7.4) at 30 °C (Table 1, entry 9). Polymerization proceeded rapidly with quantitative monomer conversion achieved within 1 h. Notably, the initialization period observed in the P*t*BuSMAnh-*b*-PNAM block copolymerization was absent (Figure 1). It is well-established that acrylamide monomers (which already exhibit comparatively higher *k*_p_ than other monomer classes) demonstrate accelerated polymerization kinetics in aqueous media, which could have resulted in elimination of the initialization period previously observed during the synthesis of P*t*BuSMAnh-*b*-PNAM in 1,4-dioxane.^36,43,44^ The resulting P*t*BuSMA-*b*-PNAM block copolymer was analyzed *via* DOSY NMR spectroscopy and SEC (Figure 2C). The DOSY NMR spectrum showed signals characteristic of P*t*BuSMA and PNAM with similar diffusion coefficients (D = 3.26 × 10^–7^ cm^2^/s), but additional signals with a larger diffusion coefficient (*D* = 4.33 × 10^–7^ cm^2^/s) were also observed, corresponding to predominantly P*t*BuSMA protons (*H*_b_ & H_c_). The P*t*BuSMA-*b*-PNAM eluogram had shifted to lower elution volumes compared to the P*t*BuSMA macro-CTA, suggesting successful chain extension, but with significant broadening of the molecular weight distribution (*Đ* = 1.55). As demonstrated *vide supra*, the 3,5-dimethylpyrazole Z-group and P*t*BuSMAnh macro-R-group mediate the well-controlled polymerization of NAM, albeit with an initialization period. It is plausible that this initialization period facilitates ‘single-unit monomer insertion’ (SUMI) of NAM into the P*t*BuSMAnh macro-CTA, such that all chains grow simultaneously and rapidly once SUMI is complete. For the aqueous system, this initialization period is absent which could enhance hybrid behavior during the RAFT-mediated block copolymerization.^45,46^ This would result in BCP chains growing to different extents during the block copolymerization, yielding the P*t*BuSMAnh-rich material observed during DOSY NMR spectroscopic analysis and the broad molecular weight distribution obtained *via* SEC analysis.

### Hydrolysis of Block Copolymers

The amphiphilic PSMAnh-*b*-PNAM and P*t*BuSMAnh-*b*-PNAM block copolymers (entry 5 and 8, Table 1) underwent alkaline hydrolysis to yield their DHBC derivatives, PSMA-*b*-PNAM and P*t*BuSMA-*b*-PNAM respectively. The alkaline hydrolysis of PSMAnh-type copolymers generally involves heating the copolymer (in the form of a powder or precipitate) in a basic aqueous environment. As the MAnh repeat units along the copolymer backbone are hydrolyzed to their MAc form, the solubility of the copolymer in water increases, resulting in the gradual dissolution of the solid polymer into the aqueous medium. This process generally utilizes high reaction temperatures and long reaction times. However, the permanently hydrophilic PNAM component of the amphiphilic BCPs prevents the precipitation of the copolymers upon addition to the alkaline aqueous phase, thereby maximizing the exposure of the anhydride-containing block to the aqueous environment. Consequently, complete hydrolysis of the copolymer was achieved within 0.5 h (assessed *via* ATR-FTIR spectroscopy, Figure S8), applying relatively mild reaction conditions. PSMA-*b*-PNAM and P*t*BuSMA-*b*-PNAM were purified *via* dialysis to remove excess NaOH and lyophilized, to facilitate assessment of DHBC conformation upon exposure to various stimuli.

### Solution Properties

Copolymer hydrophobicity, ionization state and charge density are some factors that have a significant influence on the conformation of PSMA-type copolymers in aqueous media and their interactions with phospholipid bilayers.^5^ PSMA is an amphiphilic copolymer constituting hydrophilic MAc and hydrophobic STY repeat units, while P*t*BuSMA is a more hydrophobic analogue of PSMA due to the *tert*-butyl moieties along the backbone. The water-solubility of PSMA and P*t*BuSMA amphiphilic copolymers arises from the negatively charged carboxylate functional groups of the MAc units along the polymer backbone. These functional groups are partially ionized at neutral *p*H (PSMA MAc unit *p*K_a1_ = 4.5, *p*K_a2_ = 8.9 and P*t*BuSMA MAc unit *p*K_a1_ = 5.6, *p*K_a2_ = 8.0, respectively).^42^ The ionization state and charge density of PSMA and P*t*BuSMA can be tuned by variation of *p*H or the concentration of divalent cations (Mg^2+^/Ca^2+^) in solution. Increasing the ionization state of the MAc repeat units promotes intra- and intermolecular electrostatic repulsion and the adoption of a random coil conformation, while a decrease in ionization state and charge density has been shown to promote a collapsed coil conformation in water. The latter scenario has been shown to promote higher efficiency insertion of the copolymer chains into lipid membranes, allowing for the successful formation of SMALPs.^5^ The DHBC architecture of PSMA-*b*-PNAM and P*t*BuSMA-*b*-PNAM increases the complexity of the copolymer’s aqueous solution behavior. Furthermore, polymers with a block copolymer architecture, and additionally those assembled into ordered macromolecular structures such as micelles, have not been used for the solubilization of membrane proteins. The generation of Coulombic interactions between the PSMA/P*t*BuSMA block with simple multivalent metal ions or a second cationic polymer can promote the self-association of DHBCs, and the subsequent formation of HPICs. It has been established that the formation and overall stability of the HPIC is largely dependent on the nature of the metal ions and complexing functions of the copolymers, although no direct experimental evidence describing the correlation between HPIC stability and complexation strength has been reported to the best of our knowledge.^21^ The interactions between Ca^2+^/Mg^2+^ and copolymers with pendant carboxylate groups (e.g., PAA-*b*-PEG/PMA-*b*-PEG) are generally too weak to produce dense and well-defined HPICs, even with the addition of a stoichiometric equivalent of the cation exceeding that of the anionic carboxylate moieties.^21,47,48^ It should also be noted that PNAM does not form stable complexes with divalent cations *via* its amide functional groups and therefore should exclusively perform the role of stabilizing constituent during HPIC formation.^49^

Thus, the aggregation behavior of PSMA-*b*-PNAM and P*t*BuSMA-*b*-PNAM, with decreasing *p*H or in the presence of Mg^2+^/Ca^2+^, was investigated. To assess the behavior of the DHBCs synthesized in this study in aqueous media, solutions of PSMA-*b*-PNAM and P*t*BuSMA-*b*-PNAM (3 mg/mL) were prepared, titrated with MgCl_2_, CaCl_2_ or HCl and the hydrodynamic diameter of the polymer assessed *via* DLS as a function of the charge ratio Z (eq S1, Figure 3C). Similar titrations were performed for PSMA and P*t*BuSMA macro-CTAs to provide comparative values for divalent cation/*p*H tolerance.

**Figure 3:**
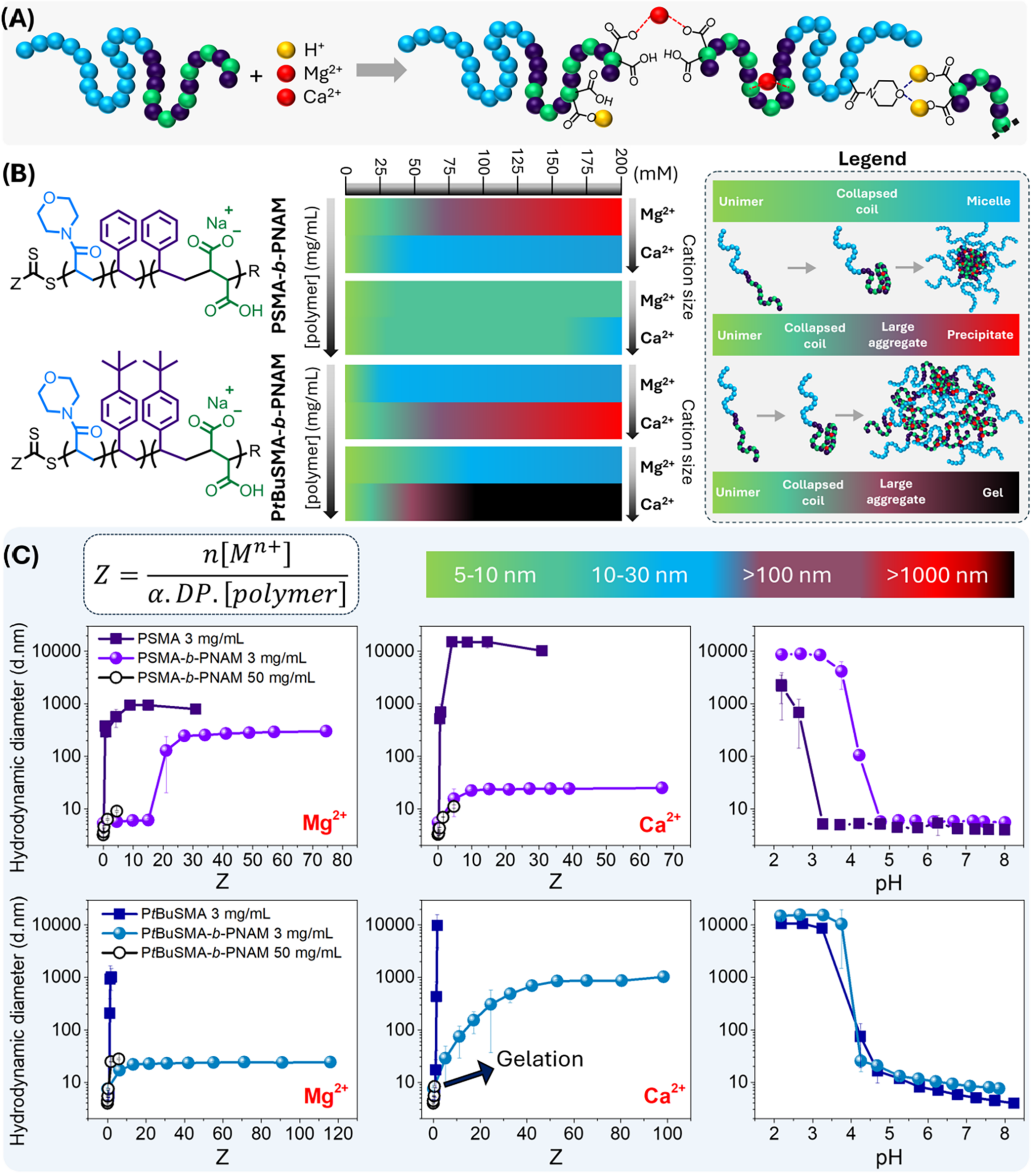
(A) Graphical representation of inter- and intramolecular chelation between PSMA-*b*-PNAM/P*t*BuSMA-*b*-PNAM and M^2+^, as well as hydrogen bond formation between carboxylic acid and morpholine pendant groups. (B) Graphical summary of the aggregation state of the DHBCs at varying polymer concentration (3 or 50 mg/mL), varying M^2+^ (Mg^2+^ or Ca^2+^) and varying M^2+^ concentration (0–200 mM). (C) Titration of the P*t*BuSMA/PSMA macro-CTAs and corresponding P*t*BuSMA-*b*-PNAM/PSMA-*b*-PNAM block copolymers, with CaCl_2_ (0.4 M), MgCl_2_ (0.4 M) and HCl (1.0 M). The hydrodynamic diameter of the copolymers at varying concentration was assessed *via* DLS and expressed as a function of the Z ratio (eq S1).

### *p*H Tolerance

The block copolymers were titrated with HCl, which increases the hydrophobicity of the PSMA/P*t*BuSMA block *via* protonation of the carboxylate groups along the backbone, effectively yielding an amphiphilic block copolymer. Decreasing the *p*H from around 8.0 causes a gradual increase in the hydrodynamic diameter of P*t*BuSMA-*b*-PNAM and PSMA-*b*-PNAM until *p*H 4.3 and *p*H 5.4 are reached respectively, at which point self-assembly is not observed but rather a rapid increase in hydrodynamic diameter occurs as the BCPs precipitate (Figure 3C). In its protonated form, the PSMA-*b*-PNAM and P*t*BuSMA-*b*-PNAM block copolymers constitute a hydrogen bond donor block (PSMA/P*t*BuSMA) and a hydrogen bond acceptor block (PNAM). Therefore, a significant proportion of carboxylic acid functional groups at low *p*H promote hydrogen bonding interactions between the polyacid and PNAM block (as demonstrated in Figure 3A), allowing for interpolymer complexation (a phenomenon which has been exploited in the synthesis of interpenetrating polymer networks).^50,51^

### PSMA-*b*-PNAM/M^2+^ Solution Behavior

The PSMA macro-CTAs synthesized in this study exhibited an initial hydrodynamic diameter of 4 nm and maintained their solubility in aqueous media upon titration with Mg^2+^/Ca^2+^ up to *Z* = 0.50–0.75 (1.5–3.0 mM Mg^2+^/Ca^2+^), after which precipitation of the polymer occurred (Figure 3C). The PSMA-*b*-PNAM block copolymer exhibited improved tolerance toward Mg^2+^ (up to Z = 15 or 24 mM Mg^2+^), as only a slight increase in hydrodynamic diameter was observed (5.3–6.1 nm) (Figure 3C). Increasing the block copolymer concentration from 3 to 50 mg/mL decreased the Z ratio, resulting in improved Mg^2+^ tolerance (up to 200 mM Mg^2+^ or *Z* = 4.6), where only a slight increase in hydrodynamic diameter occurred (3.2–8.9 nm). The titration of PSMA-*b*-PNAM with Mg^2+^ at *p*H 12.7 (Figure S10) resulted in an increase in hydrodynamic diameter from 5.2 to 22.5 nm (*Z* > 4 or [Mg^2+^] > 20 mM). At *p*H > 9 all carboxylic acid functional groups are deprotonated, promoting chelation of Mg^2+^ within a singular MAc unit and intramolecular chelation (MAc units within the same copolymer chain), as opposed to intermolecular chelation (prevalent at *p*H ≈ 7.8). It is possible that this efficiently increases the amphiphilic characteristics of the DHBC, facilitating self-assembly of PSMA-*b*-PNAM/Mg^2+^ into larger ordered structures such as micelles (ca. 23 nm).

PSMA-*b*-PNAM underwent an increase in hydrodynamic diameter from 5.5 to 22.1 nm (*Z* = 0–10, [Ca^2+^] = 0–24 mM) upon titration with Ca^2+^ at *p*H 7.8 (Figure 3C). Micelle formation was observed from *Z* = 10 where no further change in hydrodynamic diameter (ca. 24 nm) was observed up to *Z* = 67 ([Ca^2+^] = 150 mM). Additionally, micellization was obtained with a *Z* value of 4 at *p*H 12.3, yielding micelles with hydrodynamic diameters of approximately 26 nm (Figure S10).

The self-assembly of the PSMA-*b*-PNAM DHBC was assessed *via* ^1^H NMR spectroscopy. The ratio of integrated signals characteristic of PSMA and PNAM protons (*H*_f_ & *H*_a_ respectively, Figure 4A,E) was used to describe the extent of micellization. Varying [Mg^2+^] between 0–200 mM (Z = 0–4.6) for PSMA-*b*-PNAM yielded minimal reduction in signal intensity for PSMA protons (8%, *H*_f_, Figure 4A). Additionally, an increase in hydrodynamic diameter from 5.1 to 13.8 nm was observed, suggesting that PSMA-*b*-PNAM/Mg^2+^ adopted a collapsed coil conformation and formed colloidally stabilized aggregates and not micelles.^52^ Varying [Ca^2+^] from 0–100 mM (*Z* = 0–2.3) resulted in 69% shielding of PSMA protons and an increase in hydrodynamic diameter from 5.1 (PSMA-*b*-PNAM) to 36.9 nm (PSMA-*b*-PNAM/Ca^2+^), suggesting that micelles had formed. Doubling [Ca^2+^] (*Z* = 4.6) facilitated 88% reduction in signal intensity for *H*_f_ (Figure 4D,E) with a slight increase in hydrodynamic diameter (39.1 nm) observed.

**Figure 4:**
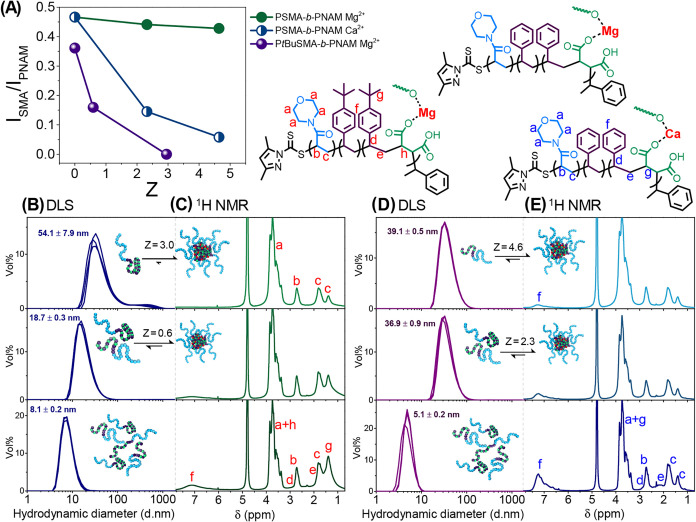
(A) Quantitative analysis of block copolymer micellization (*via* ^1^H NMR spectroscopic analysis), where the integral ratio between PSMA/P*t*BuSMA and PNAM protons is expressed as a function of the charge ratio Z. (B) DLS (measurements in triplicate) and (C) ^1^H NMR spectroscopic analysis of P*t*BuSMA-*b*-PNAM at 50 mg/mL in D_2_O (150 mM NaCl, *p*H ≈ 7) with increasing [Mg^2+^]. (D) DLS and (E) ^1^H NMR spectroscopic analysis of PSMA-*b*-PNAM at 50 mg/mL in D_2_O (150 mM NaCl, *p*H ≈ 7) with increasing [Ca^2+^].

TEM analysis was performed to assess the morphology and size of the PSMA-*b*-PNAM/M^2+^ complexes. As suggested by the preceding DLS and ^1^H NMR analysis, PSMA-*b*-PNAM/Mg^2+^ at 200 mM Mg^2+^ (*Z* = 4.6) had indeed not formed micelles, rather diffuse material lacking a defined structure was observed in all assessed micrographs (Figure 5A). TEM analysis of PSMA-*b*-PNAM/Ca^2+^ at 200 mM Ca^2+^ (*Z* = 4.6), however, exhibited some evidence of spherical structures with diameters of approximately 20 nm (slightly smaller than the hydrodynamic diameter of around 25 nm determined *via* DLS).

**Figure 5:**
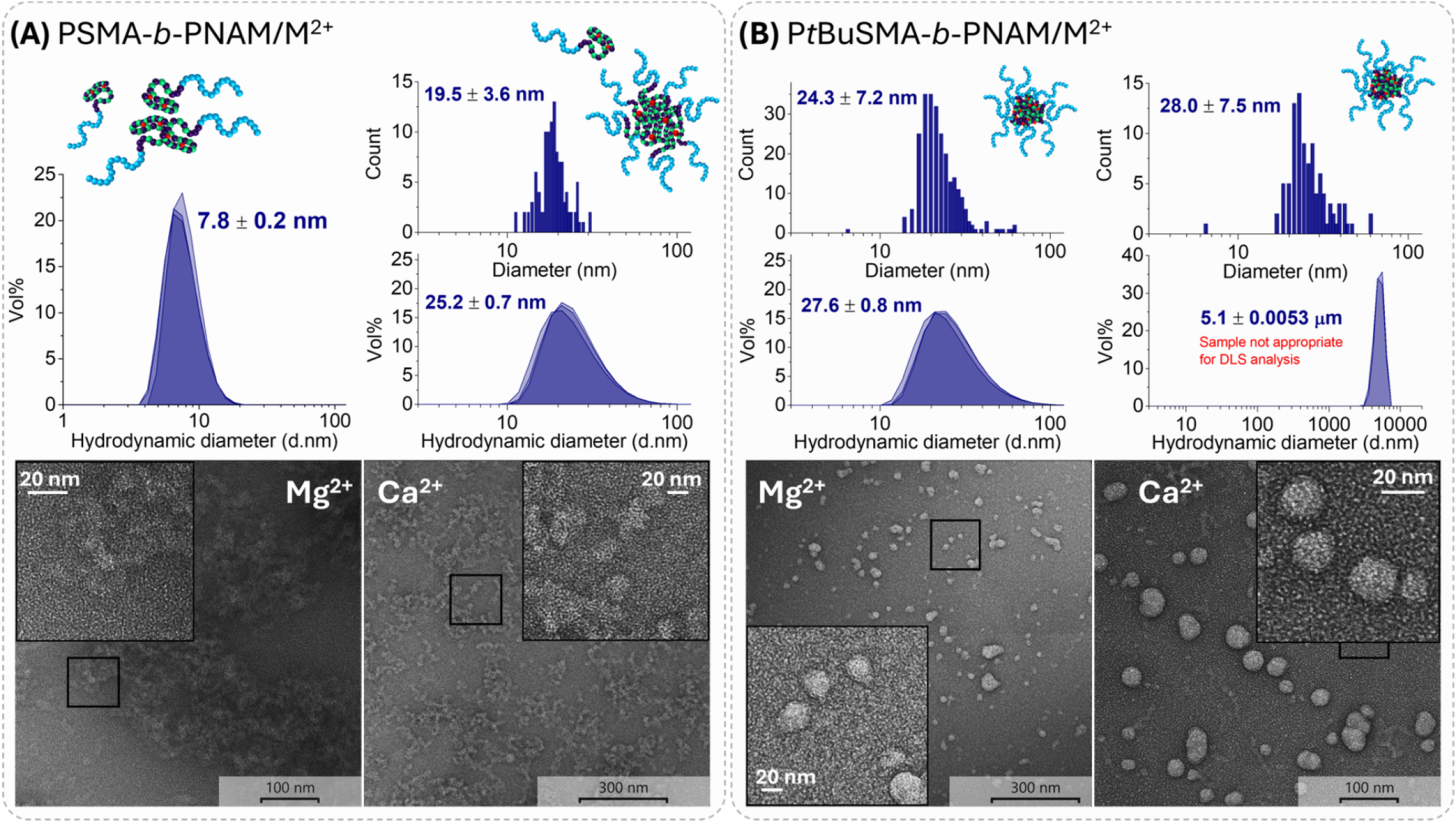
TEM and DLS analyses for (A) PSMA-*b*-PNAM/M^2+^ complexes at 200 mM Mg^2+^/Ca^2+^ (Z = 4.6) and (B) P*t*BuSMA-*b*-PNAM/M^2+^ complexes at 200 mM Mg^2+^ (*Z* = 5.9) and 64 mM Ca^2+^ (*Z* = 1.9). DHBC/M^2+^ complexes were prepared at 50 mg/mL, analyzed *via* DLS (in triplicate) and subsequently diluted to 5 mg/mL for TEM analyses.

The disparate solution behavior of the PSMA-*b*-PNAM/Mg^2+^ and PSMA-*b*-PNAM/Ca^2+^ complexes could be a result of the differences in coordination behavior and ionic radius of the divalent cation. Calcium prefers higher coordination numbers (up to 8) compared to magnesium (up to 6), the average metal–oxygen distances are approximately 2.4 Å for Ca^2+^ and 2.05 Å for Mg^2+^ and furthermore, the binding of Mg^2+^ to carboxyl groups occurs in a monodentate manner while Ca^2+^ can bind in a mono- and bidentate manner.^53^ The coordination behavior of Ca^2+^ has therefore been linked to the formation of HPICs with much larger structures compared to metal ions with smaller coordination numbers.^22^ Layrac et al. demonstrated the effect of metal coordination behavior on HPIC formation using PAA-*b*-PAM/Mg^2+^, PAA-*b*-PAM/Al^3+^ or PAA-*b*-PAM/(Mg^2+^-Al^3+^) complexes.^54^ Micelles were not formed using PAA-*b*-PAM/Mg^2+^ due to the lack of a sufficiently insoluble Mg-PAA complex phase, but the inclusion of Al^3+^ allowed for successful micelle formation as PAA was more selective for the trivalent cation.^54^ Rothnie and co-workers have previously demonstrated that increasing divalent cation size decreases the solubility of PSMA in water.^9^ They found that SMALPs created with PSMA (having a STY:MAc composition of 2:1) had the highest tolerance to the smallest divalent cation assessed (Mg^2+^ < 4 mM with an ionic radius of 0.65 Å). SMALPs exhibited decreased tolerance toward larger cations such as Ca^2+^ (>1 mM, with an ionic radius of 0.99 Å), where Co^2+^ and Zn^2+^ yielded immediate destabilization of SMALPs.^9^ Furthermore, isothermal titration calorimetry experiments performed for HPICs based on Ca^2+^, Sr^2+^ or Ba^2+^ suggest that the binding between the cations and polymers is an entropy driven process relating to the amount of water released from hydration shells during binding, where Ca^2+^ exhibited the highest enthalpy and entropy of binding.^21,22,55^ To the best of our knowledge similar experiments have not been conducted for Mg^2+^ however, it is established that magnesium exhibits stronger binding to water compared to calcium, which would theoretically result in lower entropic gain during HPIC formation.^53^ This might further validate the differences observed during the formation of PSMA-*b*-PNAM/Mg^2+^ HPICs compared to PSMA-*b*-PNAM/Ca^2+^ HPICs.

It is therefore plausible that PSMA-*b*-PNAM/Mg^2+^ complexes synthesized in this study, produced *via* the interaction of the smaller Mg^2+^ cation with the PSMA block, constitute a PSMA-Mg^2+^ phase which is still too hydrophilic to promote self-assembly into ordered structures, rather promoting a collapsed coil conformation and the formation of colloidally stabilized aggregates if an appropriately high enough polymer concentration is employed. However, interaction between PSMA and the larger Ca^2+^ cation, with higher coordination number, allows for the formation of an insoluble PSMA-Ca^2+^ phase and self-assembly into micelles.

### P*t*BuSMA-*b*-PNAM/M^2+^ Solution Behavior

PSMA-*b*-PNAM exhibited improved tolerance to divalent cations under appropriate conditions, which has promising implications for the use of this DHBC in membrane protein research. However, RAFT-synthesized PSMA has a strongly alternating character which creates a SMALP-forming block that is too hydrophilic to efficiently solubilize phospholipid membranes.^5^ The more hydrophobic P*t*BuSMA analogue has been shown to effectively mediate the solubilization of membrane proteins with higher efficiency than PSMA.^7^

P*t*BuSMA displayed an initial hydrodynamic diameter of 4.6 nm under the applied experimental conditions. Upon increasing the concentration of Mg^2+^ and Ca^2+^, a slight increase in hydrodynamic diameter (6–9 nm) is observed up to *Z* = 1, followed by a rapid increase in hydrodynamic diameter (>1 μm at *Z* > 1), while the copolymer also precipitated out of solution (Figure 3C).

For P*t*BuSMA-*b*-PNAM (3 mg/mL), an increase in [Mg^2+^] (*Z* = 0–6) resulted in a gradual increase in hydrodynamic diameter as P*t*BuSMA-*b*-PNAM/Mg^2+^ complexes began to form, and subsequently larger sized aggregates (ca. 24 nm) were observed. Similar results were obtained for P*t*BuSMA-*b*-PNAM/Mg^2+^ complexes at 50 mg/mL (Figure 3B,C). Unlike the aggregation behavior of PSMA-*b*-PNAM/Mg^2+^, the P*t*BuSMA-*b*-PNAM/Mg^2+^ complexes at *p*H 12.3 did not result in the formation of micelles, but rather larger aggregates (68–146 nm at *Z* = 0–34, Figure S10).

The aggregation behavior of P*t*BuSMA-*b*-PNAM/Mg^2+^ HPICs was also assessed *via* ^1^H NMR spectroscopy. Increasing [Mg^2+^] to 20 mM (*Z* = 0.6) resulted in P*t*BuSMA-*b*-PNAM/Mg^2+^ HPICs with a hydrodynamic diameter of ca. 19 nm and a corresponding decrease in the signal intensity for protons characteristic of P*t*BuSMA (*H*_f_ & *H*_g_, Figure 4C), where the integral ratio suggested micellization of 55%. Upon increasing the concentration of Mg^2+^ further (100 mM, *Z* = 3.0), the sample constitutes 100% P*t*BuSMA-*b*-PNAM/Mg^2+^ micelles (ca. 54 nm, Figure 4B), which is characterized by the complete disappearance of P*t*BuSMA protons (*H*_f_ & *H*_g_).

To further assess the morphology and size of the structures formed during the aggregation of P*t*BuSMA-*b*-PNAM/Mg^2+^ complexes, TEM analysis was conducted. TEM micrographs for P*t*BuSMA-*b*-PNAM/Mg^2+^ (at 50 mg/mL, *Z* = 5.9) exhibited spherical structures approximately 24 nm in diameter, only slightly smaller than the hydrodynamic diameter determined *via* DLS (ca. 27 nm). Therefore, at neutral *p*H, P*t*BuSMA-*b*-PNAM/Mg^2+^ complexes produced *via* the interaction of Mg^2+^ with the comparatively hydrophobic and sterically bulky P*t*BuSMA block, constituted a P*t*BuSMA-Mg^2+^ phase which is sufficiently insoluble in aqueous media, promoting self-assembly into micellar structures.

An increase in [Ca^2+^] caused a gradual increase in the hydrodynamic diameter of P*t*BuSMA-*b*-PNAM/Ca^2+^ complexes (at 3 mg/mL) and thereafter precipitation of the polymer, where similar results are obtained at *p*H 4.8, 8.0, and 12.2 (Figure S10). P*t*BuSMA-*b*-PNAM exhibited interesting solution behavior at elevated polymer concentration (50 mg/mL) as the system initially produced turbid solutions at low [Ca^2+^] (*Z* < 1.5), with spherical structures of 28 nm observed *via* TEM (Figure 5B), but formed a “gel-like” material at *Z* > 1.5. Therefore, the aggregation behavior of P*t*BuSMA-*b*-PNAM/Ca^2+^ (at 50 mg/mL, *Z* > 1.5) could not be analyzed appropriately *via* DLS or ^1^H NMR spectroscopy. Owing to the observed “gel-like” behavior, these materials were also probed using rheology. Amplitude sweeps, and subsequently frequency sweeps of P*t*BuSMA-*b*-PNAM/Ca^2+^ were carried out to assess the effect of polymer concentration and [Ca^2+^] on the mechanical properties of the gels, where the results are presented in Figures S14–15.

Overall, effective micellization at near neutral *p*H was achieved for P*t*BuSMA-*b*-PNAM and PSMA-*b*-PNAM using Mg^2+^ (*Z* > 6) and Ca^2+^ (*Z* > 10) respectively, whereas neutralization and precipitation of P*t*BuSMA and PSMA was observed at *Z* > 0.5–1. The formation of P*t*BuSMA-*b*-PNAM and PSMA-*b*-PNAM HPICs only occurred at much higher Z values, suggesting that the induced amphiphilicity of the P*t*BuSMA-*b*-PNAM/Mg^2+^ and PSMA-*b*-PNAM/Ca^2+^ complexes is necessary but not always sufficient to trigger self-assembly of the complexes into structures such as micelles. This phenomenon has been observed previously for PMA-*b*-PEG/Ca^2+^, PAA-*b*-PEG/Ca^2+^ and PAA-*b*-PEG/Ba^2+^ complexes.^47,48,56^ It should be noted that all DHBC titrations with Mg^2+^/Ca^2+^ at acidic *p*H (4.5–4.9) resulted in precipitation of the block copolymer. In addition to Mg^2+^/Ca^2+^ induced charge screening, the acidified medium increases the prevalence of hydrogen bonding between the PSMA/P*t*BuSMA and PNAM blocks, causing a rapid increase in hydrophobicity without added stabilization from the PNAM block.

### Solubilization of Synthetic Lipid Vesicles

The PSMA-*b*-PNAM and P*t*BuSMA-*b*-PNAM block copolymers both exhibited improved tolerance to high concentrations of Mg^2+^/Ca^2+^ compared to their PSMA/P*t*BuSMA macro-CTAs, as the block copolymers were amenable to self-assembly into HPICs (Figure 3B). This potentially offers the opportunity to solubilize membrane proteins at higher divalent cation concentrations than generally reported for PSMA/P*t*BuSMA. To assess the utility of copolymers with a DHBC architecture as new tools for membrane protein research, the solubilization of DMPC vesicles (as model cell membranes) was conducted. Thereafter, SMALPs created using PSMA-*b*-PNAM and P*t*BuSMA-*b*-PNAM were exposed to ‘salty’ media ([M^2+^] = 0–0.4 M) to assess whether the added steric stabilization afforded by the DHBCs aids in maintaining SMALP stability when electrostatic stabilization is reduced.

Dose response experiments were conducted to ascertain the appropriate concentration of block copolymer required for the effective solubilization of DMPC vesicles (5 mg/mL in Tris HCl buffer, 50 mM, *p*H = 7.6). Within the range assessed (5.0–25.0 mg/mL), a P*t*BuSMA-*b*-PNAM concentration of 25.0 mg/mL (corresponding to a [P*t*BuSMA]:[DMPC] ratio of 2.2:1) yielded the fastest solubilization kinetic profile (Figure S17). Additionally, solubilization using the block copolymer resulted in a similar kinetic profile to that using the P*t*BuSMA macro-CTA, which would suggest that the PNAM block does not have a deleterious effect on interactions of the alternating copolymer segment with the lipid bilayer. DLS analysis showed that increasing the concentration of polymer resulted in the creation of SMALPs with decreasing hydrodynamic diameter (24–9 nm), where P*t*BuSMA-*b*-PNAM SMALPs were slightly larger compared to P*t*BuSMA SMALPs (Figure S18).

P*t*BuSMA-*b*-PNAM solutions (50 mg/mL in Tris HCl buffer) were prepared at varying [Mg^2+^] (0–200 mM), added to DMPC vesicles (10 mg/mL in Tris HCl buffer) and the solubilization efficiency assessed *via* turbidimetry, DLS and TEM (Figure 6A–E). As demonstrated *vide supra*, P*t*BuSMA-*b*-PNAM at [Mg^2+^] < 20 mM adopts a collapsed coil confirmation, whereas [Mg^2+^] > 20 mM promotes the aggregation of P*t*BuSMA-*b*-PNAM/Mg^2+^ complexes into micelles (Figure 3B). Therefore, the solubilization of DMPC vesicles in Figure 6 is facilitated by unimeric P*t*BuSMA-*b*-PNAM/Mg^2+^ complexes between 0–8 mM Mg^2+^, or a proportion of P*t*BuSMA-*b*-PNAM/Mg^2+^ micelles at 32–100 mM Mg^2+^. Solubilizations conducted using unimeric species exhibited faster solubilization kinetics, with a complete decrease in optical density observed within 5 min. P*t*BuSMA-*b*-PNAM at 32 mM Mg^2+^ yielded a slower kinetic profile with a significant drop in optical density only observed after 1 h. Increasing [Mg^2+^] to 100 mM (*Z* = 6), which corresponds to a sample which is predominantly micelles (Figure 4), resulted in only a 50% drop in O.D._600 nm_ after 1.4 h. Despite the significant differences in kinetic profiles between 0–100 mM Mg^2+^, all samples yielded particles with hydrodynamic diameters between 8.9–10.3 nm, significantly smaller than those observed for DMPC vesicles (403 ± 53 nm, Figure 6D), suggesting that SMALPs were successfully formed. TEM analysis of the crude solubilization solutions showed that homogeneous nanodiscs indeed were formed, with morphologies similar to those observed for SMALPs resulting from traditional PSMA-type polymers which lack the block copolymer architecture. The average diameter of the P*t*BuSMA-*b*-PNAM SMALPs was extracted from the micrographs, where the representative samples at 32 mM and 100 mM Mg^2+^ were determined to have nanodisc diameters of 14–17 nm (Figure 6E). Solubilization experiments were also conducted using P*t*BuSMA-*b*-PNAM at varying [Ca^2+^], however light scattering analyses were limited to 0–8 mM Ca^2+^ as the block copolymer solution prepared at 64 mM (for a final [Ca^2+^] = 32 mM) undergoes gelation before it can be efficiently mixed with the DMPC vesicle solution (Figure S19).

**Figure 6:**
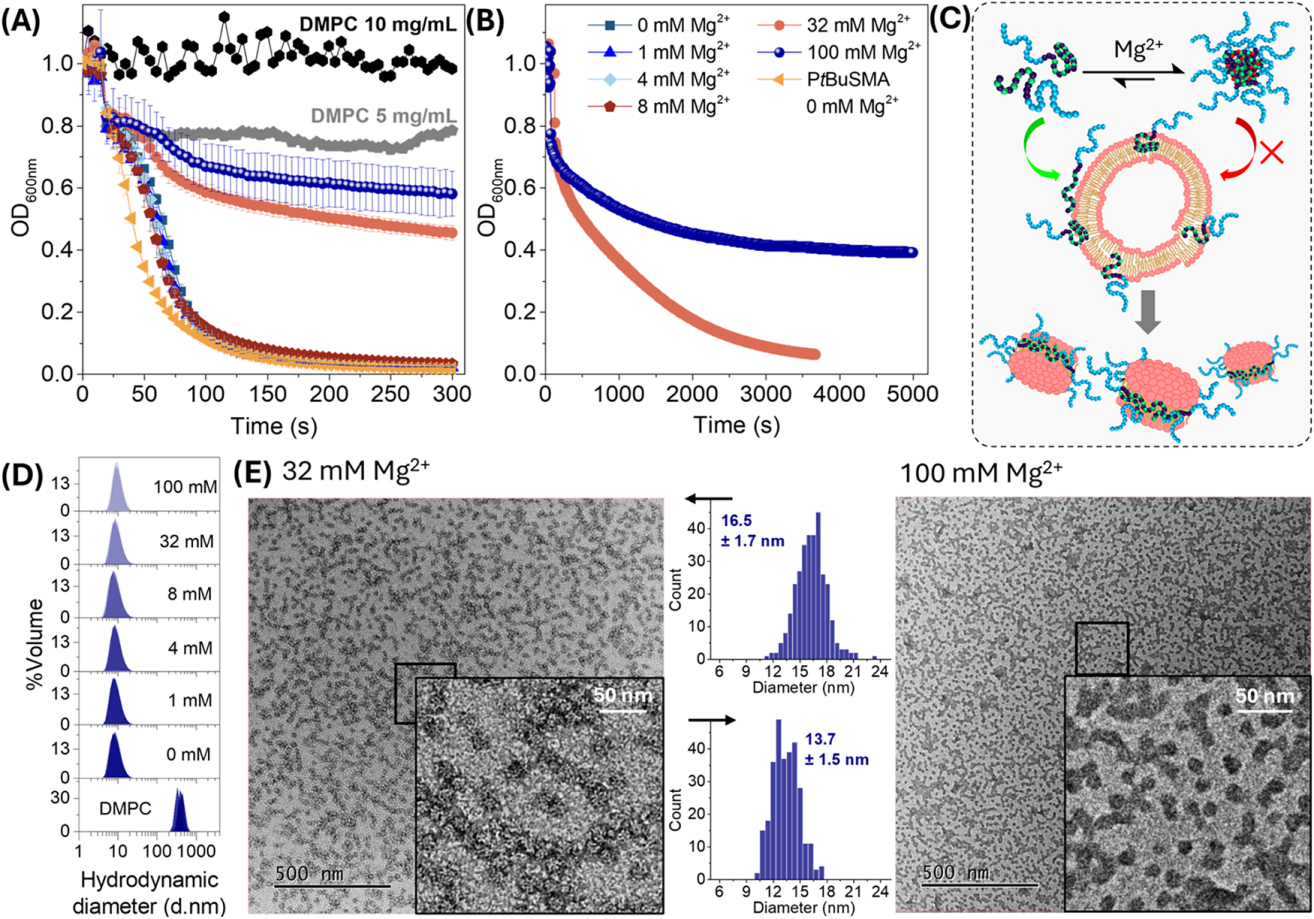
(A) Turbidimetric analysis (600 nm at 25 °C, 5 min) for the solubilization of DMPC vesicles (final concentration of 5 mg/mL in Tris HCl buffer, 50 mM, *p*H 7.8) using P*t*BuSMA-*b*-PNAM (final concentration of 25 mg/mL in Tris HCl buffer) at varying concentrations of Mg^2+^ (final concentrations between 0–100 mM). B) The same turbidimetric analysis performed in (A) but with a 1.4 h incubation time. (C) Graphic representation of free chain vs micelle and how this equilibrium affects the solubilization process. (D) DLS analysis of P*t*BuSMA-*b*-PNAM SMALPs. (E) TEM analysis of P*t*BuSMA-*b*-PNAM SMALPs formed at 32–100 mM Mg^2+^.

HPICs exhibit high colloidal stability and generally resist aggregation with time, lyophilization and resuspension, or an increase in ionic strength (monovalent ions).^20,21,57,58^ Additionally, HPICs are generally hypothesized to be kinetically trapped and not at a thermodynamic equilibrium as they do not undergo instantaneous aggregate dissociation upon dilution.^21,22^ However, some exchange of polymer between aggregate and solution is possible depending on the nature of the metal, copolymer complexing capacity as well as external parameters such as *p*H.^59^ It is likely that P*t*BuSMA-*b*-PNAM/Mg^2+^ micelles have negligible interaction with the lipid bilayer, as the micelle corona constitutes PNAM, which does not interact with the membrane (Figure S16). It is therefore plausible that some exchange of P*t*BuSMA-*b*-PNAM/Mg^2+^ occurs between micelles and the bulk aqueous phase making these copolymers available for interaction with the lipid bilayer (Figure 6C). The equilibrium between unimeric P*t*BuSMA-*b*-PNAM/Mg^2+^ complexes and micelles is therefore imposed upon the solubilization process, resulting in a significant reduction in solubilization kinetics. Increasing [Mg^2+^] from 32 to 100 mM favors micelle formation and results in a further reduction in solubilization kinetics (Figure 6B,C), but ultimately leads to the successful formation of SMALPs (Figure 6E).

Similar solubilization experiments were conducted using PSMA-*b*-PNAM at varying [Mg^2+^] and [Ca^2+^]. PSMA is considerably more hydrophilic than P*t*BuSMA and is generally known to have poor efficiency in the solubilization of lipid membranes. The chelation of Mg^2+^/Ca^2+^ to the PSMA component of the block copolymer screens the anionic carboxylate groups, creating hydrophobic domains along the backbone which have unfavorable interactions with water and comparably favorable interactions with the lipid acyl chains. The formation of PSMA-*b*-PNAM/M^2+^ complexes directs the polymer to adopt a collapsed coil conformation (up to 200 mM Mg^2+^, Figure 3B) and additionally to self-assemble into micellar structures (up to 200 mM Ca^2+^, Figure 3B). PSMA-*b*-PNAM is too hydrophilic at 0 mM Mg^2+^/Ca^2+^ and therefore exhibited poor solubilization efficiency, but utilizing [Mg^2+^/Ca^2+^] ≥ 4 mM (*Z* > 0.2) effectively increased the hydrophobicity of the PSMA block, resulting in the successful formation of SMALPs as evidenced by DLS (6–27 nm) and TEM (21–24 nm) (Figure 7B,C). The solubilization efficiency of PSMA-*b*-PNAM at 0 mM Mg^2+^/Ca^2+^ could also be improved by protonating the MAc residues of the PSMA block *via* acidification of the solution (*p*H 4.5–5.2), yielding SMALPs with hydrodynamic diameters ranging between 9–40 nm as determined *via* DLS (Figures S20–21).

**Figure 7:**
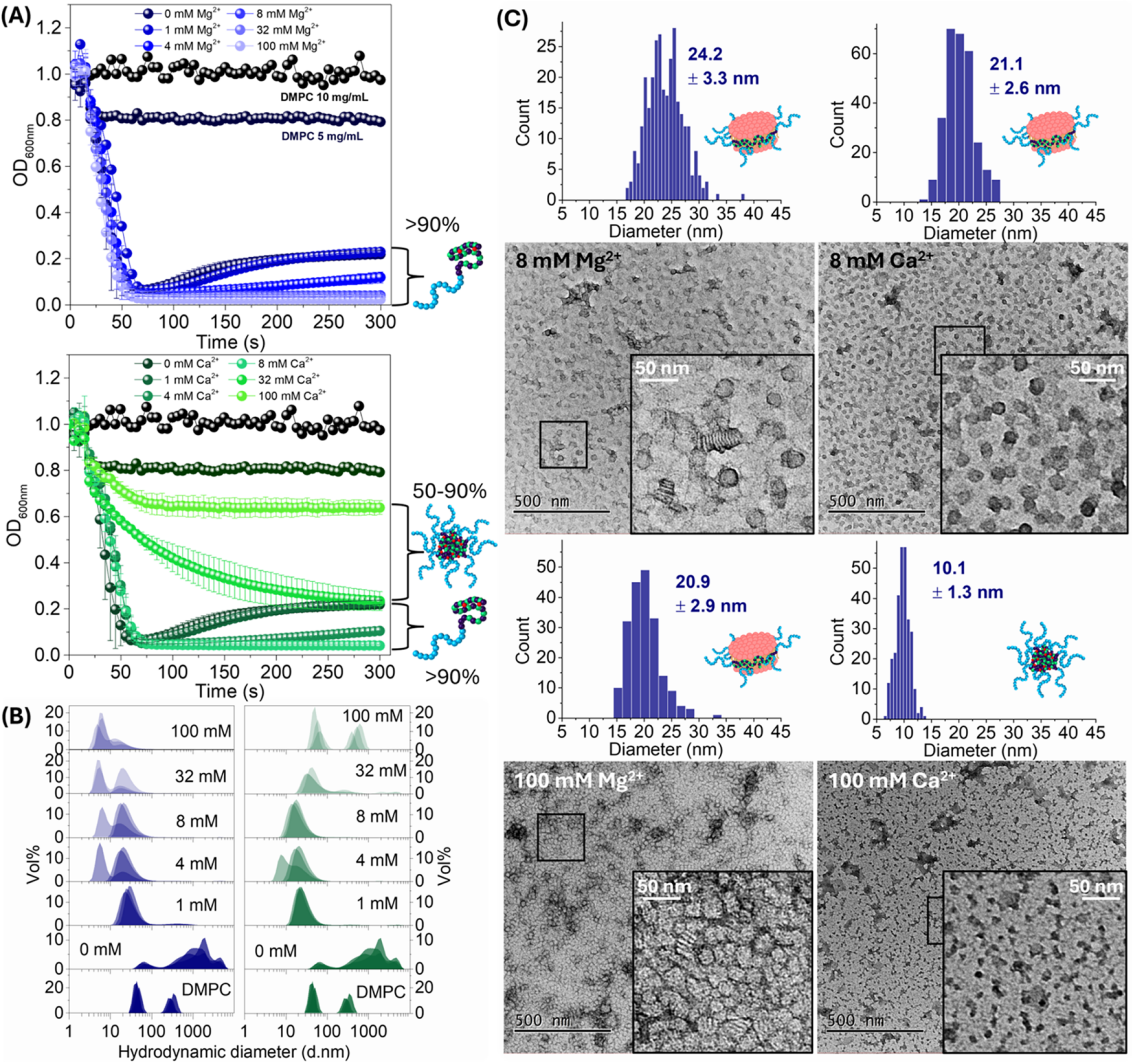
(A) Turbidimetric analysis (600 nm at 25 °C, 5 min) for the solubilization of DMPC vesicles using PSMA-*b*-PNAM (final concentration of 25 mg/mL in Tris HCl buffer) at [Mg^2+^] = 0–100 mM (left) or at [Ca^2+^] = 0–100 mM (right). An approximation of free chain vs micelle abundance is based on data presented in Figure 4. (B) DLS analysis of resulting solubilization solutions, for Mg^2+^ (left) and Ca^2+^ (right) experiments, respectively. (C) TEM micrographs for PSMA-*b*-PNAM SMALPs formed at 8/100 mM Mg^2+^/Ca^2+^ as well as PSMA-*b*-PNAM micelles obtained at 100 mM Ca^2+^.

While PSMA-*b*-PNAM at [Mg^2+^] ≥ 32 mM (Z > 1.5) exhibited fast solubilization kinetics and yielded SMALPs of ca. 21 nm in diameter, [Ca^2+^] ≥ 32 mM (Z > 1.5) promoted micellization (50–90%) which significantly reduced solubilization efficiency. TEM micrographs obtained for the solubilization of DMPC vesicles using PSMA-*b*-PNAM at 100 mM Ca^2+^ (*Z* = 4.6) did not indicate the formation of nanodiscs but rather displayed dense spherical structures which are likely to represent polymer micelles (ca. 10 nm) (Figure 7C). DMPC solubilizations conducted using P*t*BuSMA-*b*-PNAM (at 100 mM Mg^2+^) or PSMA-*b*-PNAM (at 100 mM Ca^2+^) are undertaken with polymer samples which predominantly constitute micelles (>90%) (Figure 4). However, the former solubilization proceeds at much higher efficiency than the latter. This might suggest that more stabilized PSMA-*b*-PNAM/Ca^2+^ micelles were formed compared to P*t*BuSMA-*b*-PNAM/Mg^2+^ micelles, which has the potential to decrease the concentration of unimeric PSMA-*b*-PNAM/Ca^2+^ available for solubilization. Alternatively, P*t*BuSMA-*b*-PNAM/Mg^2+^ could interact with the lipid membrane more efficiently than PSMA-*b*-PNAM/Ca^2+^, despite the improved hydrophobicity of PSMA-*b*-PNAM/Ca^2+^ complexes. In that case the depletion of free chains *via* interactions with the membrane would be slower, resulting in the prevalence of PSMA-*b*-PNAM/Ca^2+^ micelles which ultimately do not interact with the lipid membrane.

Ultimately, the hydrophobicity of the PSMA block could be tuned to significantly improve its solubilization efficiency, up to concentrations of Mg^2+^/Ca^2+^ that are generally unsuitable for PSMA but made possible through utilization of PSMA-*b*-PNAM. This provides a facile approach to tuning the amphiphilicity of the solubilizing block without requiring pre/post-polymerization modifications. It should be noted that analogues of PSMA-*b*-PNAM would have vastly different solution behavior in the presence of Mg^2+^/Ca^2+^, as demonstrated by the inclusion of P*t*BuSMA-*b*-PNAM in this study. Any variations in the metal-type or metal-interactive block which affect the coordination behavior with M^2+^, are likely to result in significant alterations in the self-assembly behavior of the DHBC. These variations could include changes in the amphiphilicity of the block or the steric bulk in the vicinity of the coordination site.

### Steric Stabilization of SMALPs

SMALPs which are created using traditional PSMA-type copolymers are stabilized against aggregation *via* Coulombic repulsion of their charged surfaces and anionic polymer belt. Coulombic screening is reduced *via* the interplay of two nonexclusive mechanisms, i.e., an increase in the ionic strength of the aqueous medium and association of counterions (e.g., Mg^2+^ or Ca^2+^) with the carboxylate groups on the PSMA-type copolymer.^60^ Excessive Coulombic screening can result in aggregation and precipitation of the polymer and lipid content of the SMALP.^9^ It is hypothesized that SMALPs created using the DHBCs in this study would afford steric stabilization in addition to electrostatic stabilization, resulting in maintained SMALP stability at high divalent cation concentrations. Steric stability of SMALPs is particularly desirable for researchers investigating MPs such as ABC transporters, as the assays employed for assessing MP activity use high [Mg^2+^].^8^

Therefore, SMALP stock solutions were prepared *via* solubilization of DMPC vesicles using P*t*BuSMA, P*t*BuSMA-*b*-PNAM or PSMA-*b*-PNAM/Mg^2+^. These SMALPs were exposed to increasing concentrations of M^2+^ (0–400 mM) and changes in the hydrodynamic diameter of the SMALPs assessed *via* DLS (Figure 8). While P*t*BuSMA and P*t*BuSMA-*b*-PNAM SMALPs could be formed at 0 mM M^2+^, PSMA-*b*-PNAM is too hydrophilic to facilitate SMALP formation. Thus, the stock of SMALPs was formed using PSMA-*b*-PNAM/Mg^2+^ (at 4 mM Mg^2+^).

**Figure 8:**
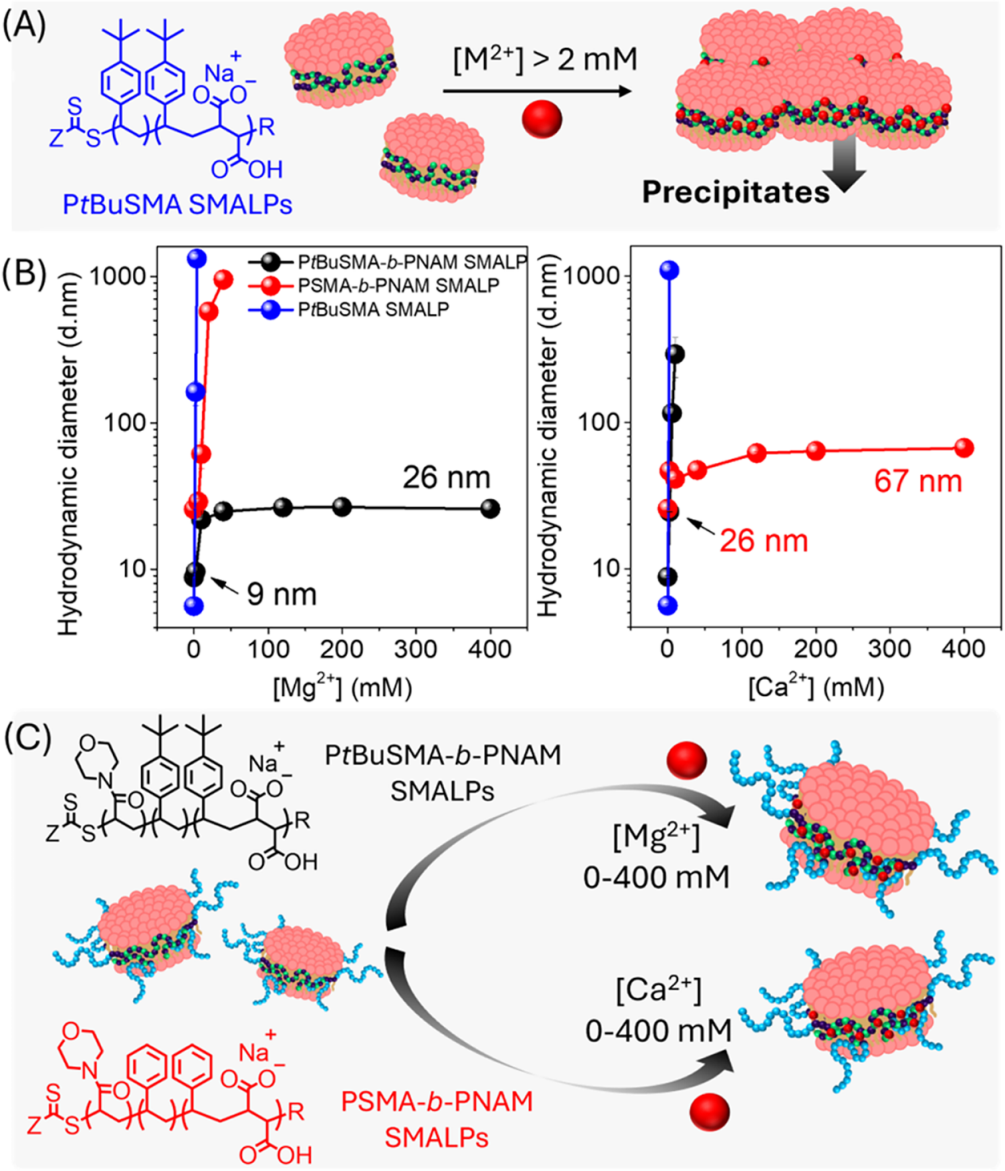
(A) Graphical representation of electrostatically stabilized SMALPs, formed using P*t*BuSMA and the effects of increasing [M^2+^]. (B) DLS analysis of SMALPs (created using P*t*BuSMA, P*t*BuSMA-*b*-PNAM and PSMA-*b*-PNAM/Mg^2+^) which were incubated at 25 °C for 14 h before exposure to increasing [M^2+^]. Individual solutions (1 mL) were prepared with increasing [M^2+^] (0–400 mM), 100 μL aliquots of the SMALP solutions added and the solution analyzed using DLS. (C) Graphical representation of electrostatically and sterically stabilized SMALPs, formed using P*t*BuSMA-*b*-PNAM and PSMA-*b*-PNAM/Mg^2+^, and the effects of increasing [M^2+^].

As expected, increasing [M^2+^] above 2 mM resulted in loss of electrostatic repulsion between P*t*BuSMA SMALPs causing a rapid increase in hydrodynamic diameter due to precipitation of lipid and copolymer (Figure 8A). P*t*BuSMA-*b*-PNAM SMALPs exhibited a slight increase in hydrodynamic diameter (9–21 nm) between 0–10 mM Mg^2+^ and thereafter stabilized at 26 nm (10–400 mM Mg^2+^), whereas increasing [Ca^2+^] caused rapid precipitation of lipid and copolymer. PSMA-*b*-PNAM/Mg^2+^ SMALPs exhibited a slight increase in hydrodynamic diameter (26–42 nm) between 0–40 mM Ca^2+^ followed by stabilization at 67 nm, whereas increasing [Mg^2+^] resulted in precipitation of lipid/copolymer. The DHBCs therefore exhibited similar solution behavior when exposed to Mg^2+^/Ca^2+^ as demonstrated with the free copolymers *vide supra*, despite their positioning at the lipid–water interface. SMALPs are not kinetically trapped structures despite their high thermodynamic and colloidal stability, but are rather highly dynamic assemblies which exhibit rapid exchange of both lipid and polymer chains.^61,62^ Lipid exchange predominantly occurs *via* collisions between particles, whereby the highly flexible nature of PSMA facilitates the fast reorganization of the polymer belt upon collision.^5,61^ Thus, SMALPs exhibit much faster lipid exchange dynamics than other membrane mimic systems.^61^ Reducing Coulombic repulsion between SMALPs, *via* increasing the concentration of Mg^2+^ and Ca^2+^, results in significantly faster collisional transfer of lipids.^60^ In the present study, the effect of increasing [Mg^2+^/Ca^2+^] is 2-fold; the chelation of the cations to the PSMA or P*t*BuSMA blocks reduces Coulombic repulsion between SMALPs and additionally alters the conformation of the copolymer at the lipid–water interface. It is plausible that this facilitates the reorganization of these lipid assemblies into slightly larger SMALPs, which are thereafter solely stabilized *via* steric repulsion if the correct combination of divalent cation and block copolymer is employed. Thus, understanding the solution behavior of the DHBCs can aid in the selection of an appropriate block copolymer to afford effective steric stabilization of SMALPs for assays that require high concentrations of divalent cations.

## Conclusions

Low *Đ* (1.13–1.24) PSMAnh-*b*-PNAM and P*t*BuSMAnh-*b*-PNAM amphiphilic block copolymers (*M*_n_ ≈ 14 500 g/mol) were synthesized successfully *via* RAFT-mediated copolymerization. Rapid alkaline hydrolysis of the block copolymers was achieved, yielding PSMA-*b*-PNAM and P*t*BuSMA-*b*-PNAM DHBCs respectively. Titration of the DHBCs (and corresponding PSMA/P*t*BuSMA macro-CTAs) with Mg^2+^/Ca^2+^ facilitated the formation of DHBC/M^2+^ hybrid polyionic complexes at *Z* > 1 (and the precipitation of the macro-CTAs at *Z* ≤ 1). The successful formation of micelles, with average hydrodynamic diameters around 24 nm was observed for P*t*BuSMA-*b*-PNAM/Mg^2+^ (*Z* > 6) and PSMA-*b*-PNAM/Ca^2+^ (*Z* > 10) at neutral *p*H, where aggregation was induced at slightly lower *Z* with application of higher DHBC concentration. It was hypothesized that the interplay of properties such as the relative size of Mg^2+^/Ca^2+^, their disparate coordination behavior with water and carboxylate functional groups and the relative inherent amphiphilicity and steric bulkiness of the PSMA/P*t*BuSMA block, significantly affected the extent of HPIC formation and aggregation. The DHBCs were subsequently utilized for the solubilization of DMPC vesicles. P*t*BuSMA-*b*-PNAM/M^2+^ complexes (0–100 mM M^2+^) efficiently solubilized the lipid bilayer into SMALPs (evidenced by light scattering techniques and TEM), however aggregated P*t*BuSMA-*b*-PNAM/Mg^2+^ (as micelles) and P*t*BuSMA-*b*-PNAM/Ca^2+^ (as a gel) resulted in slower solubilization kinetics and inhibition of solubilization, respectively. PSMA-*b*-PNAM did not yield effective solubilization of DMPC vesicles, as the PSMA segment is considered too hydrophilic for appropriate intercalation into the lipid bilayer. However, the formation of PSMA-*b*-PNAM/M^2+^ complexes (4–100 mM Mg^2+^ and 4–8 mM Ca^2+^) enhanced the amphiphilicity of the PSMA block and significantly improved the solubilization efficiency of the copolymer. The PSMA-*b*-PNAM/Ca^2+^ complex at 100 mM Ca^2+^ was determined to predominantly consist of micelles which resulted in complete loss of solubilization efficiency. It was hypothesized that PSMA-*b*-PNAM/Ca^2+^ micelles have higher stability than P*t*BuSMA-*b*-PNAM/Mg^2+^ micelles, resulting in negligible exchange of the copolymer with the aqueous medium and therefore yielding insufficient free copolymer chains for disruption of the lipid bilayer. With the application of the appropriate conditions, the DHBCs synthesized in this study overcame a significant limitation of PSMA-type copolymers encountered during the solubilization of membrane proteins. Investigations regarding the utility of these DHBCs for the solubilization of ABC transporters, which require high Mg^2+^ concentrations to function optimally, are ongoing. Nevertheless, this work has demonstrated that DHBCs are an interesting class of copolymers with untapped potential for membrane protein research endeavors.

## Supplementary Material



## Notes

The SI constitutes the experimental section, ^1^H NMR spectra, SEC eluograms, ATR-FTIR spectra, TEM micrographs, DLS data and turbidimetric data (PDF)

## †

Leibniz-Institut für Polymerforschung Dresden e.V., Hohe Straße 6, D-01069 Dresden, Germany

## Abbreviations

STY/*t*BuSTY: styrene/4-*tert*-butylstyrene
MAnh: maleic anhydride
MAc: maleic acid
DMPC: 1,2-dimyristoyl-*sn*-glycero-3-phosphocholine
PSMAnh: poly­(styrene-*alt*-maleic anhydride)
P*t*BuSMAnh: poly­(4-*tert*-butylstyrene-*alt*-maleic anhydride)
PSMA-*b*-PNAM: poly­(styrene-*alt*-maleic acid)-*block*-poly­(4-acryloylmorpholine)
P*t*BuSMA-*b*-PNAM: poly­(4-*tert*-butylstyrene-*alt*-maleic acid)-*block*-poly­(4-acryloylmorpholine)
SMALP: poly­(styrene-*alt*-maleic acid) lipid particle
