# Supplementary material for: Aqueous Solution Behavior of Poly(styrene-alt-maleic acid)‑b‑poly(N‑acryloylmorpholine) Double Hydrophilic Block Copolymers in the Absence and Presence of Divalent Cations and Phospholipids
Source: Macromolecules. 2025 Jun 24;58(13):6804–19. doi: 10.1021/acs.macromol.5c01099 (PMC12257593; doi:10.1021/acs.macromol.5c01099)
Supplement: Supplementary file 1 [file ma5c01099_si_001.pdf]

Supplementary Information

Aqueous solution behavior of poly(styrene-*alt*-maleic acid)-*b*-poly(*N*-acryloylmorpholine) double hydrophilic block copolymers in the absence and presence of divalent cations and phospholipids

Lauren E. Ball †, Michael-Phillip Smith, Bennie Motloung, Rueben Pfukwa* and Bert Klumperman*.*

Department of Chemistry and Polymer Science, University of Stellenbosch, Matieland 7602, South Africa.

† Leibniz-Institut für Polymerforschung Dresden e.V., Hohe Straße 6, D-01069 Dresden, Germany.

Corresponding authors:

Lauren E. Ball: ball@ipfdd.de

Bert Klumperman: bklump@sun.ac.za

Rueben Pfukwa: rueben@sun.ac.za

Materials

Styrene (STY, Merck, ≥ 99%, stabilized with *tert*-butylcatechol) and 4-*tert*-butyl-styrene (*t*BuSTY, Merck, 93%, stabilized with *tert*-butylcatechol) were eluted from an aluminum oxide (Merck, activated basic, Brockmann I) column, prior to use. Maleic anhydride (MAnh, Merck, 99%) and 1,3,5-trioxane (Merck, 99%) were recrystallized from distilled chloroform, filtered and dried under vacuum or alternatively sublimed as needed. 4-Acryloylmorpholine (NAM, stabilized with 1000 ppm monomethyl ether hydroquinone, Merck, 97%) was vacuum distilled before use. Azobisisobutyronitrile (AIBN, Merck, 98%) was recrystallized from anhydrous methanol, filtered and dried under vacuum. THF was pre-dried over KOH pellets, filtered and distilled over sodium/benzophenone. A PBS solution (*p*H = 7.4) constituting 0.01 M phosphate buffer, 0.0027 M potassium chloride and 0.137 M sodium chloride was prepared *via* dissolution of one PBS tablet (Merck) in 200 mL deionized (DI) H_2O. Tris HCl buffer (1 M, UltraPure™, Invitrogen) was diluted to 50 mM (*p*H 7.6) using DI water. 1,2-Dimyristoyl-*sn*-glycero-3-phosphocholine (DMPC, powder, Merck),

$MgCl_2·6H_2O$ (Merck, ≥ 99%), $CaCl_2$ (Merck, ≥ 99%), KOH (Merck, 90%, flakes), CS_2 (Kimix, 99%), 3,5-dimethylpyrazole (Merck, 99%), 1-bromoethyl benzene (Merck, 97%), Na_2SO_3 (Merck, ≥98%), *t*BuOOH (Merck, 70 wt.% in H_2O), Na_2CO_3 (Merck, ≥98%), 1,4-dioxane (Merck, anhydrous, 99.8%), acetone (Merck, ≥ 99.5%), *N*,*N*-dimethylformamide (DMF, Merck, anhydrous, 99.8%), NaOH (Merck, pellets for analysis) and 3500 MWCO SnakeSkin™ dialysis tubing (Thermo Fisher Scientific) were used as received.

Experimental methods

Synthesis of PNAM

The CTA, 1-phenylethyl 3,5-dimethyl-1*H*-pyrazole-1-carbodithioate (PEPC), was synthesized according to procedures reported in a previous study, with similar yield and purity.[1] NAM (1.0 g, 7.2 mmol), PEPC (49 mg, 0.18 mmol), AIBN (5.6 mg, 34 µg), 1,3,5-trioxane (19 mg, 0.22 mmol) and 1,4-dioxane (3.5 mL) were added to a three-neck round-bottom flask (20 mL), fitted with a magnetic stirrer bar, rubber septum and bubbler. The solution was sparged with dry argon for 30 min, an aliquot (0.1 mL) withdrawn using a degassed syringe, and subsequently immersed in an oil bath preheated at 60 °C for 24 h. The solution was cooled and a final kinetic sample withdrawn, before the polymer was isolated *via* precipitation in pentane (80 mL). The precipitate was centrifuged (4500 rpm, 3 min), redissolved in DCM (5 mL) and precipitated in pentane (80 mL). This process was repeated thrice and the isolated polymer dried under vacuum at ambient temperature for 24 h. Kinetic samples were diluted using $(CD_3)_2CO$ (0.5 mL) and analyzed *via* ^1H NMR spectroscopy to determine monomer conversion. The isolated PNAM was characterized *via* SEC, ^1H NMR spectroscopy and ATR-FTIR spectroscopy.

Synthesis of PSMAnh/P*t*BuSMAnh macro-CTAs

PSMAnh and P*t*BuSMAnh macro-CTAs were synthesized according to previously reported procedures.[1] Considering P*t*BuSMAnh as an exemplary copolymer, *t*BuSTY (12 g, 74 mmol, 1.5 eq relative to MAnh), MAnh (5.0 g, 51 mmol), PEPC (0.70 g, 2.6 mmol), AIBN (8.3 mg, 0.5 mmol), 1,3,5-trioxane (0.1 g, 1.1 mmol) and 1,4-dioxane (60 mL) were added to a three-neck round-bottom flask (100 mL) fitted with a magnetic stirrer bar, rubber septum and bubbler. The solution was sparged with dry argon for 45 min, an aliquot withdrawn (ca. 0.1 mL) with a degassed syringe, and the flask subsequently immersed in an oil bath preheated at 60 °C for 22 h. Kinetic samples (ca. 0.5 mL) were withdrawn at specified time intervals using a degassed syringe and subsequently diluted in $(CD_3)_2CO$ for ^1H NMR spectroscopic analysis or dried under

vacuum and redissolved at 2 mg/mL (in THF, 5% AcOH) for SEC analysis. The reaction mixture was cooled and diluted with acetone (10–20 mL) and the polymer precipitated in pentane (1 L). P*t*BuSMAnh was isolated *via* vacuum filtration and dried under vacuum at ambient temperature for 24 h. The polymer was characterized *via* SEC, ^{1}H NMR and ATR-FTIR spectroscopy. The synthesis of PSMAnh was conducted using a similar procedure but alternatively employed 1.3 eq of STY relative to MAnh.

Hydrolysis of PSMAnh/P*t*BuSMAnh macro-CTAs

The procedure for alkaline hydrolysis of PSMAnh and P*t*BuSMAnh macro-CTAs was adapted from previously reported methods. Briefly, PSMAnh (2.1 g, 0.36 mmol) was dissolved in acetone (8 mL) and added to a solution of NaOH (0.63 g, 16 mmol, 1.0 eq relative to MAnh units) in DI water (21 mL). This caused immediate precipitation of the copolymer, but with continued stirring at 60 °C for 20 h, dissolution of the copolymer was achieved due to successful transformation of MAnh repeat units to MAc repeat units along the copolymer backbone. The solution was cooled and transferred to dialysis tubing (3500 MWCO) and dialyzed against DI water for 2–3 days (with the exchange of dialysis water occurring 2–3× daily). The PSMA copolymer was isolated *via* lyophilization and characterized using ATR-FTIR and ^{1}H NMR spectroscopy. For the alkaline hydrolysis of P*t*BuSMAnh, the copolymer (5.4 g, 0.89 mmol) was dissolved in acetone (18 mL) and added to a solution of NaOH (1.3 g, 31 mmol, 1.5 eq relative to MAnh units) in DI water (250 mL). The suspension was heated at 60 °C for 48 h; refluxed at 100 °C for 2 h and subsequently cooled for transferal to dialysis tubing (3500 MWCO). The solution was dialyzed against DI water for 2–3 days (with water changes occurring 2–3× daily). The P*t*BuSMA copolymer was isolated *via* lyophilization and characterized using ATR-FTIR and ^{1}H NMR spectroscopy.

Synthesis of PSMAnh-*b*-PNAM and P*t*BuSMAnh-*b*-PNAM

An exemplary protocol for the synthesis of P*t*BuSMAnh-*b*-PNAM is as follows. P*t*BuSMAnh (M_n^{theo} = 6 100 g/mol, 1.0 g, 0.16 mmol), NAM (1.4 g, 10 mmol), AIBN (2.8 mg, 17 μmol), 1,3,5-trioxane (21 mg, 0.23 mmol) and 1,4-dioxane (8.8 mL) were added to a three-neck round-bottom flask (20 mL), fitted with a magnetic stirrer bar, rubber septum and bubbler. The solution was sparged with dry argon for 30 min, an aliquot (0.1 mL) withdrawn with a degassed syringe, and the flask subsequently immersed in an oil bath preheated at 60 °C for 23 h. The solution was cooled, a kinetic sample withdrawn (0.1 mL) and the solution diluted with DCM (2–5 mL). The block copolymer was isolated *via* precipitation in pentane (80 mL) followed by centrifugation (4 500 rpm, 3 min). The polymer pellet was dissolved in DCM (5 mL); the

precipitation procedure repeated twice more and subsequently the copolymer was dried under vacuum at ambient temperature for 24 h. PSMAnh-*b*-PNAM was synthesized using the same protocol but alternatively employing the PSMAnh macro-CTA. All block copolymers were characterized *via* SEC, ATR-FTIR, ^{1}H NMR and DOSY NMR spectroscopy.

Synthesis of P*t*BuSMA-*b*-PNAM

Alternatively, the block copolymerization was conducted in aqueous media, employing the hydrolyzed P*t*BuSMA macro-CTA. P*t*BuSMA (M_n^{theo} = 6 600 g/mol, 1.0 g, 0.15 mmol), NAM (1.3 g, 9.2 mmol), *t*BuOOH (2.7 mg, 30 µmol), DMF (13 mg, 0.18 mmol) and PBS (*p*H 7.4, 11.5 mL) were added to a three-neck round-bottom flask (20 mL) fitted with a magnetic stirrer bar, rubber septum and bubbler. The solution was deoxygenated *via* five freeze-pump-thaw cycles and the flask subsequently backfilled with argon. Na_2SO_3 (4.0 mg, 0.30 µmol) and PBS (1 mL) were added to a separate glass vial (5 mL) fitted with rubber septum; the solution sparged with argon for 10 min and subsequently added to the polymerization mixture using a degassed syringe. The flask was immersed in an oil bath preheated at 30 °C for 3.5 h and the contents of the flask subsequently transferred to dialysis tubing (3500 MWCO). The block copolymer was dialyzed against DI water for 24 h and isolated *via* lyophilization.

Hydrolysis of block copolymers

P*t*BuSMAnh-*b*-PNAM (48 g, 3.3 mmol) dissolved in THF (160 mL) was added to a preheated solution (60 °C) of NaOH (3.3 g, 68 mmol, 1 eq relative to MAnh units) in DI water (320 mL) and stirred at 60 °C for 30 min. Upon addition of the polymer, the solution immediately exhibited an increased opacity and within 10 s cleared, suggesting rapid hydrolysis of the P*t*BuSMAnh block. The solution was allowed to stir at 60 °C for 10 min before a 2 mL sample was withdrawn and dried under vacuum at ambient temperature. Within 0.5 h of the BCP's first contact with the alkaline aqueous phase, the dried BCP sample was analyzed *via* ATR-FTIR spectroscopy to confirm that successful hydrolysis of MAnh repeat units had occurred. The reaction mixture was cooled, transferred to dialysis tubing (3500 MWCO) and dialyzed against DI water for 2–3 days (with water changes occurring 2–3× daily). The hydrolyzed P*t*BuSMA-*b*-PNAM copolymer was isolated *via* lyophilization and characterized using ATR-FTIR, ^{1}H NMR and DOSY NMR spectroscopy. PSMAnh-*b*-PNAM was hydrolyzed using the same protocol, but *via* the addition of PSMAnh-*b*-PNAM (8.8 g, 0.62 mmol) dissolved in THF (30 mL) to a preheated solution of NaOH (0.87 g, 20 mmol, 1 eq relative to MAnh units) in DI water (58 mL).

Titration of block copolymers

To assess the *p*H and Mg^{2+}/Ca^{2+} tolerance of the DHBCs, 5 mL solutions of PSMA, P*t*BuSMA, PSMA-*b*-PNAM and P*t*BuSMA-*b*-PNAM were prepared at 3 mg/mL (in 50 mM Tris HCl buffer for divalent cation titrations or in 150 mM NaCl solution for acid titrations) and titrated with HCl (0.1 M), $MgCl_2$ (0.4 M) or $CaCl_2$ (0.4 M). Titrations were conducted using a MPT-2 Multi-Purpose Titrator fitted to a Zetasizer Nano series Nano-ZS DLS, to assess changes in hydrodynamic diameter with decreasing *p*H or increasing divalent cation concentration, where the resulting data was processed using Malvern Zetasizer software (version 8.02). Samples prepared at 50 mg/mL were manually titrated and the hydrodynamic diameter of the polymer assessed *via* DLS, as high polymer concentrations were incompatible with the functioning of the auto-titrator. Titration data was subsequently represented as the variation in hydrodynamic diameter as a function of Z ratio (defined in **Equation S1**).

$$Z = \frac{n[M^{n+}]}{\alpha \cdot DP \cdot [polymer]}$$ **Equation S1.**

Z is defined as the ratio of charges between cationic Ca^{2+}/Mg^{2+} and the anionic carboxylate groups of the DHBCs, where n is the valency of the cation, $[M^{n+}]$ and [polymer] are the concentration of the cation and block copolymer respectively, α is the charge of a PSMA or P*t*BuSMA block repeat unit and DP is the degree of polymerization of the PSMA or P*t*BuSMA block.

^{1}H NMR spectroscopic micelle analysis

DHBC stock solutions were prepared by dissolving P*t*BuSMA-*b*-PNAM (0.15 g, 10 μmol) and PSMA-*b*-PNAM (0.25 g, 17 μmol) in D_2O (150 mM NaCl) at 50 mg/mL. Stock solutions (0.4 M) of Mg^{2+}/Ca^{2+} were prepared by dissolving $MgCl_2 \cdot 6H_2O$ (81 mg, 0.40 mmol) and $CaCl_2$ (22 mg, 20 mmol) in 1 mL and 0.5 mL D_2O (150 mM NaCl) respectively. P*t*BuSMA-*b*-PNAM/Mg^{2+} (0–100 mM Mg^{2+}), PSMA-*b*-PNAM/Mg^{2+} (0–200 mM Mg^{2+}) or PSMA-*b*-PNAM/Ca^{2+} (0–200 mM Ca^{2+}) complexes/micelles were prepared by mixing the polymers at specific M^{2+} concentrations (with Z ratios varying from 0–4.6), the hydrodynamic diameter of the particles assessed *via* DLS and 0.5 mL of the solution was subsequently analyzed *via* ^{1}H NMR spectroscopy (600 MHz Bruker).

Solubilization of DMPC lipid vesicles

DMPC vesicles were prepared at 10 mg/mL in Tris HCl buffer (50 mM) *via* sonication of the DMPC suspension for 0.25–0.5 h. The vesicle solution (10 μL) was diluted in Tris HCl buffer (990 μL) and the

hydrodynamic diameter of the vesicles assessed using DLS, before utilization in solubilization experiments. A fresh batch of DMPC vesicles was prepared as needed, prior to each solubilization experiment. For solubilization experiments conducted at variable divalent cation concentrations, polymer solutions were prepared separately at double the required polymer/cation concentration and the hydrodynamic diameter of the polymer assessed *via* DLS prior to addition to the vesicle solution. As an exemplary solubilization experiment, a solution of [Mg^{2+}] = 400 mM (500 μL) was added to a solution of P*t*BuSMA-*b*-PNAM (50 mg, 3.4 μmol) dissolved in Tris HCl buffer (500 μL). The hydrodynamic diameter of the polymer in solution (50 mg/mL and [Mg^{2+}] = 200 mM) was assessed *via* DLS to be ca. 24 nm, therefore the conformation of the polymer was hypothesized to be a micelle structure. The DMPC vesicle solution (300 μL) was incubated at 25 °C for ca. 15 seconds in the Multiskan spectrophotometer followed by the addition of the polymer solution (300 μL) and the optical density (at 600 nm) of the solution was measured for 5 min (or up to 1.4 h). This yielded a final solution of DMPC lipid (5 mg/mL), P*t*BuSMA-*b*-PNAM (25 mg/mL) and [Mg^{2+}] = 100 mM. This solution (100 μL) was diluted in Tris HCl buffer (900 μL) and the hydrodynamic diameter and morphology of the formed particles assessed *via* DLS and TEM respectively.

Steric stabilization of SMALPs

DMPC vesicles were prepared as described *vide supra*. Polymer stock solutions were prepared at 22 mg/mL and 50 mg/mL in Tris HCl buffer (50 mM) using P*t*BuSMA or P*t*BuSMA-*b*-PNAM (i.e. 0 mM Mg^{2+} or Ca^{2+}), respectively. The SMALP solutions were prepared *via* addition of the polymer solution (500 μL) to the DMPC vesicle solution (500 μL), followed by incubation at 25 °C for 14 h. This yielded a P*t*BuSMA or P*t*BuSMA-*b*-PNAM SMALP stock solution with lipid and polymer concentrations of 5 mg/mL and 25 mg/mL (for P*t*BuSMA-*b*-PNAM) or 11 mg/mL (for P*t*BuSMA), respectively. Mg^{2+}/Ca^{2+} solutions (1 mL) were prepared in Tris HCl buffer (50 mM) at concentrations ranging between 0–400 mM. To each individual divalent cation solution, an aliquot (100 μL) of a SMALP solution was added and the change in hydrodynamic diameter of the SMALPs assessed *via* DLS. Each measurement was conducted in triplicate.

The PSMA-*b*-PNAM block copolymer was too hydrophilic to facilitate SMALP formation in the absence of divalent cations. As such, a PSMA-*b*-PNAM stock solution (50 mg/mL in Tris HCl buffer) was prepared using 8 mM Mg^{2+}. After addition to the DMPC vesicle solution, a PSMA-*b*-PNAM/Mg^{2+} SMALP stock solution was prepared with a final lipid concentration of 5 mg/mL, polymer concentration of 25 mg/mL and a

Mg^{2+} concentration of 4 mM. Aliquots of the PSMA-*b*-PNAM/Mg^{2+} SMALPs (100 µL) were added to the individual Mg^{2+}/Ca^{2+} solutions (1 mL) in a similar manner as described above and subsequently analysed *via* DLS.

Characterization

NMR spectroscopic analyses were carried out using a 400 MHz Agilent NMR Spectrometer (DOSY analyses) or a 400 MHz/600 MHz Bruker Ascend Spectrometer, specified per sample. Samples were dissolved in either $(CD_3)_2CO$ (Merck, Magni-Solv™, 99.9%), $(CD_3)_2SO$ (Merck, Magni-Solv™, 99.9%) or D_2O (Merck, Magni-Solv™, 99.9%) prior to analysis, specified per sample. All DOSY NMR spectroscopy measurements were performed at 298 K on an Agilent Inova 400 NMR spectrometer operating at 400 MHz and equipped with a two-channel multinuclear z-gradient inverse probe head capable of producing gradients in the z direction with a calibrated gradient strength of 0.00213 Gauss/cm/DAC. The DOSY spectra were acquired with the Dbppste_cc (convection compensation) pulse program from VnmrJ 4.2 topspin software. All spectra were recorded with 16–32 K time domain data points in the t2 dimension and 30 t1 increments. The gradient strength was logarithmically incremented in 30 steps from 5% up to 100% of the maximum gradient strength. All measurements were performed with a diffusion delay of around 250 ms and a gradient pulse length of 2 ms. Both of these parameters were adjusted slightly in order to ensure signal attenuation of more than 85%. The diffusion dimension of the 2D DOSY spectra was processed by a licensed Mnova 12 software package.

Size exclusion chromatography was conducted using two different systems, specified per sample. The first protocol employed LiBr stabilized DMF (2 mM, Merck, Chromosolv® Plus, for HPLC, ≥ 99.9%), with samples dissolved at 2 mg/mL prior to analysis. The samples were filtered using 0.45 µm PTFE filters (Sartorius) prior to analysis with an Agilent 1260 HPLC instrument fitted with a quaternary pump, thermostated column compartment set at 60 °C, an autosampler, a differential refractometer set at 50 °C and a diode array UV detector set at 320 nm. Columns utilized were Agilent PLgel Mixed-C (5 µm) guard column (50 × 7.5 mm i.d.) and two analytical columns (300 × 7.5 mm i.d.). The flow rate during analysis was 1.0 mL/min and the injection volume per sample was 100 µL. PMMA calibration standards (800–2.2×10^6 g/mol) as well as RAFT-synthesized SMAnh calibration standards (600–90 000 g/mol) were utilized. The second protocol employed THF (5 v/v% AcOH with 0.125% BHT, Merck, for HPLC, ≥99.9%)

with samples dissolved at 2 mg/mL and filtered using 0.45 μm RC filters (Sartorius) prior to analysis. The analysis was performed on an Agilent 1260 HPLC instrument fitted with a quaternary pump, a column compartment thermostated at 30 °C, a differential refractometer set at 30 °C and a diode array UV detector set at 254 and 320 nm. The columns utilized were two Agilent Technologies PLgel 5 Mixed-C columns (300 × 7.5 mm i.d.) and a PLgel 5 Guard column (50 × 7.5 mm i.d.). The flow rate during analysis was 1.0 mL/min and the injection volume per sample was 100 μL. The system was calibrated using low *Đ* PS calibration standards with a molar mass range of 580–2.0 $\times 10^6$ g/mol.

Attenuated total reflectance infrared (ATR-FTIR) spectroscopy was performed using a Thermo Scientific Nicolet iS10 Smart iTR, using 128 scans over the wavelength range of 600–4000 cm^{-1}, with a background spectrum (64 scans) obtained prior to each sample analyzed.

Dynamic light scattering analyses, for assessment of the polymer aggregates within polymerization mixtures, were conducted using a ZetaSizer 1000 HSa (Malvern Instruments, Malvern), fitted with a 4 mW He-Ne laser, operating at a wavelength of 633 nm and a scattering angle of 90°. Analyses were conducted using ZetaSizer Software 8.

Turbidimetry experiments were conducted using a Thermo Fisher Scientific Multiskan SkyHigh microplate spectrophotometer fitted with a xenon flash lamp and monochromator with a bandwidth less than 2.5 nm. Measurements were obtained at 600 nm, every 3 s for 5 min (or up to 1.4 h, for solubilization experiments, with samples incubated at 25 °C during analysis.

Transmission electron microscopy analyses for SMALP samples were conducted *via* dilution of samples 10× before imaging. Samples (5 μL) were applied to a glow-discharged carbon/formvar coated 300 mesh copper TEM grid (EM Resolutions, Sheffield UK) and stained with 2% uranyl acetate for 1 minute. The grids were imaged on the JEOL2100Plus electron microscope with Gatan OneView camera. TEM analyses for polymer aggregates were undertaken using a JEOL 1200 EX microscope, equipped with a Gatan Orius CCD camera, with an accelerating voltage of 120 kV utilized. Prior to imaging, a 5 mg/mL DHBC solution was prepared (×10 dilution) and 1 μL was spotted onto a copper grid. The sample was stained with a 2% uranyl acetate solution and subsequently dried at room temperature. TEM micrographs were analyzed using ImageJ software to produce size distributions (with up to ca. 200 particles measured per micrograph).

Rheological measurements were performed using an Anton Paar GmbH (MCR302) rheometer, for the determination of hydrogel mechanical properties. The instrument was fitted with a parallel plate geometry

(diameter, 25 mm) and a measuring gap of 1 mm was used. Samples were placed on the bottom plate at 25 °C, the geometry was lowered to the measuring position and the hood was subsequently lowered to enclose the sample. Amplitude sweeps were performed at a constant angular frequency of 10 rad·s^{-1} and the amplitude of oscillation was varied between 0.01 and 100%. Frequency sweeps were subsequently carried out in the 1-100 rad·s^{-1} angular frequency range, at fixed strain amplitudes. The RheoCompass software (version 1.2) was used for system control, data capturing and data processing.

Equation S2. Monomer conversion calculation for copolymerizations in Table 1 (main text).

$$\alpha = \left(1 - \frac{I_{tx}^{Monomer}}{I_{t0}^{Monomer}}\right) \times 100$$

Equation S3. For PSMAnh/P*t*BuSMAnh copolymerizations, M1/M2 refers to STY/*t*BuSTY and MAnh comonomers. For block copolymerizations M1 is NAM and the M2 term is excluded.

$$M_n^{theo} = \frac{[M1] \times MW_{M1} \times \alpha_{M1}}{[CTA]} + \frac{[M2] \times MW_{M2} \times \alpha_{M2}}{[CTA]} + MW_{CTA}$$

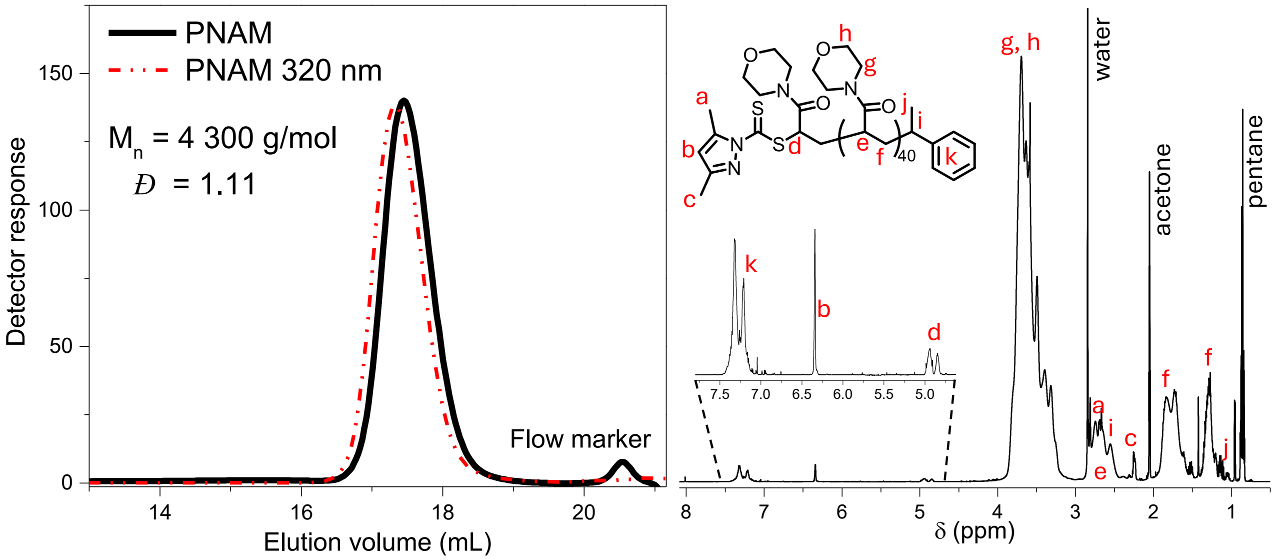

Figure S1. SEC analysis of PNAM (left) using THF (5% AcOH) as mobile phase and PS calibration standards, with RI detector response in black and UV detector response in red. ^{1}H NMR analysis (600 MHz) of PNAM in $(CD_3)_2CO$ (right). Integration of H_k (Int. = 0.93, 5 protons) compared to H_d (Int. = 0.15, 1 proton) would suggest that only 81% of the ω-chain ends were retained.

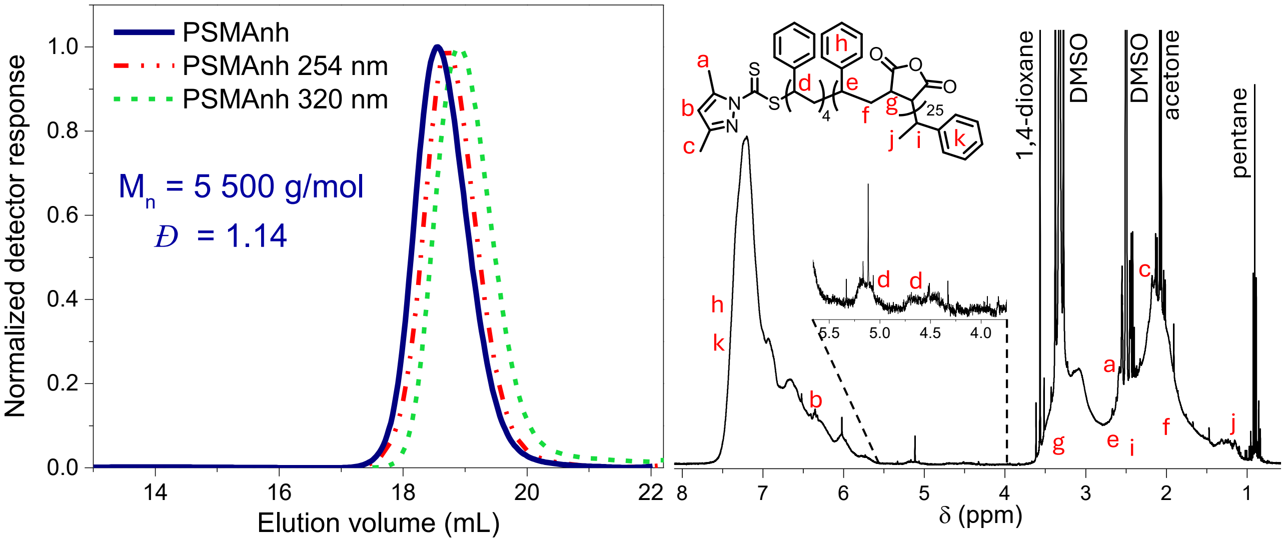

Figure S2. SEC analysis of PSMAnh (left) using THF (5% AcOH) as mobile phase and PS calibration standards, with RI detector response in dark blue and UV detector response (254 & 320 nm) overlayed in light green/red. ^{1}H NMR analysis (600 MHz) of PSMAnh in $(CD_3)_2SO$ (right).

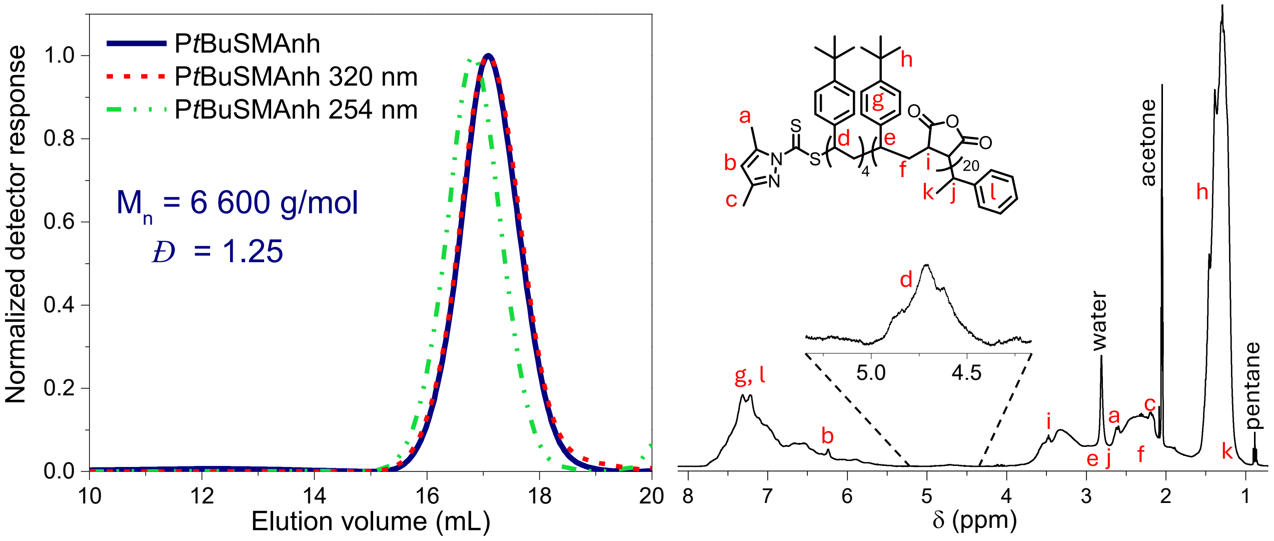

Figure S3. SEC analysis of P*t*BuSMAnh (left) using THF (5% AcOH) as mobile phase and PS calibration standards, with RI detector response in dark blue and UV detector response (254 & 320 nm) overlayed in light green/red. ^{1}H NMR analysis (600 MHz) of P*t*BuSMAnh in $(CD_3)_2CO$ (right).

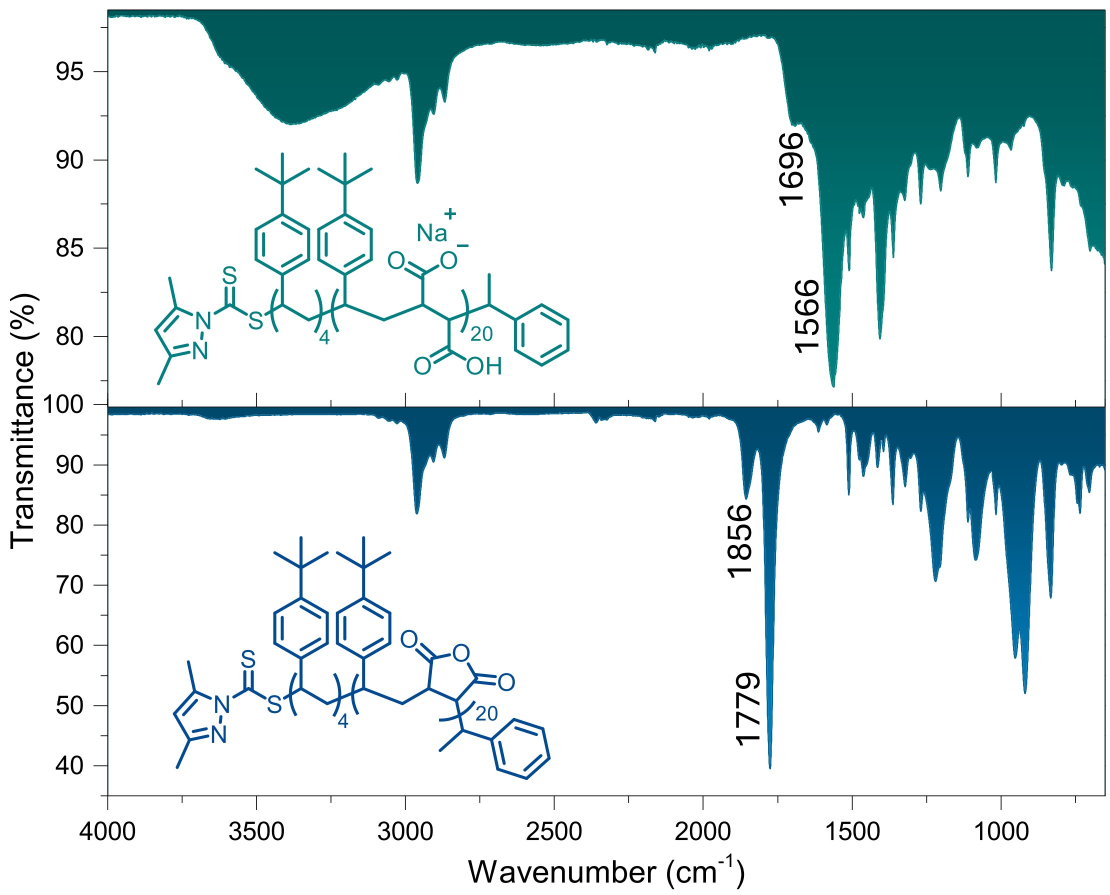

Figure S4. ATR-FTIR spectra for P*t*BuSMAnh (bottom) and P*t*BuSMA post-alkaline hydrolysis (top).

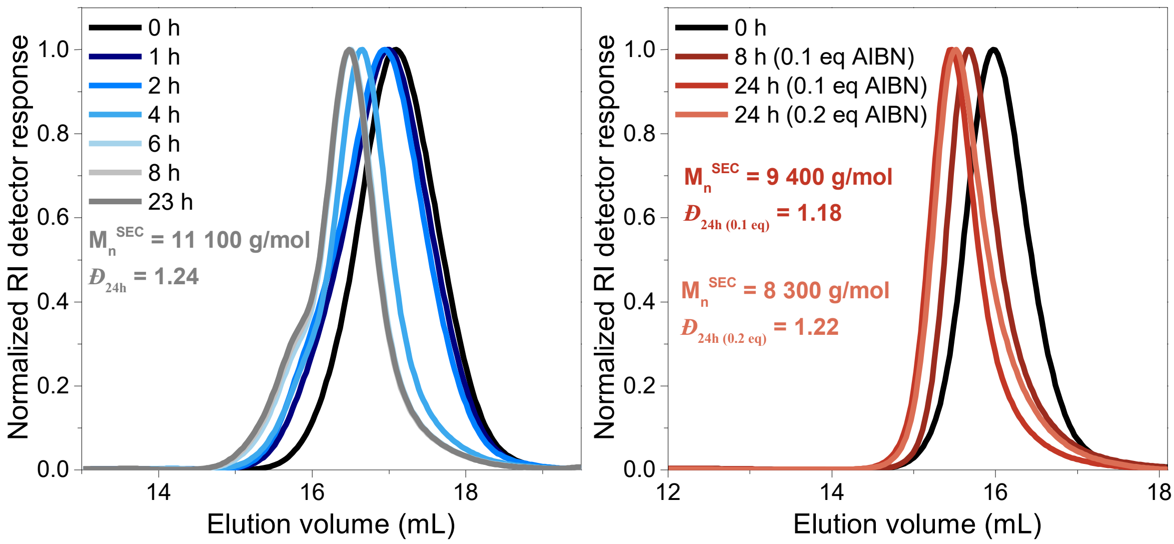

Figure S5. SEC analysis for kinetic sample withdrawn during the synthesis of P*t*BuSMAnh-*b*-PNAM (**left**, entry 8 in Table 1, main text) or the synthesis of PSMAnh-*b*-PNAM using 0.1/0.2 equivalents of AIBN (**right**, entry 6–7, Table 1, main text). Samples were analyzed using THF (5% AcOH) as the mobile phase and PS calibration standards.

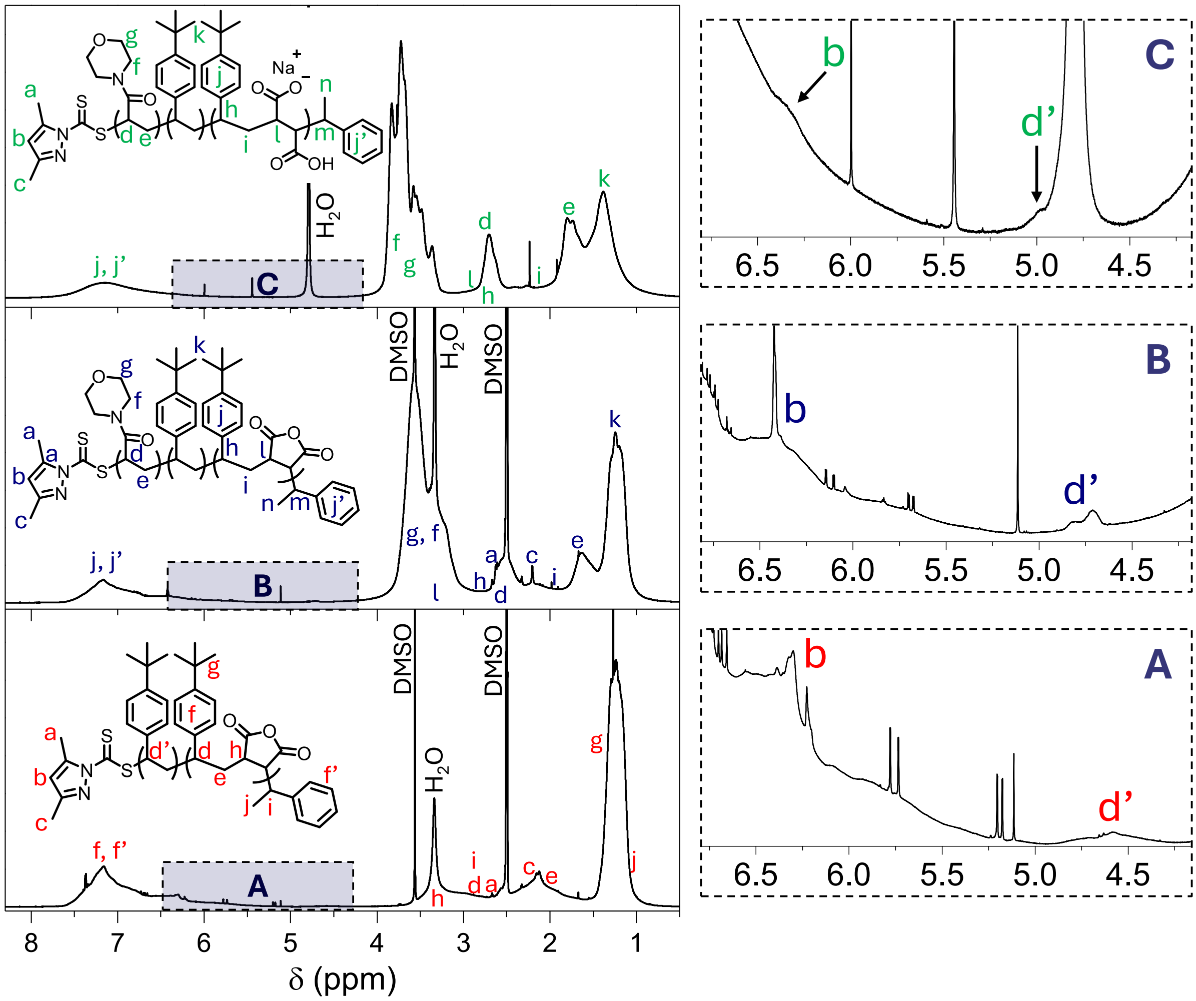

Figure S6. ^{1}H NMR spectroscopic analysis (600 MHz) of P*t*BuSMAnh (bottom), P*t*BuSMAnh-*b*-PNAM (middle) and P*t*BuSMA-*b*-PNAM (top) after BCP hydrolysis, in DMSO-d$_6$ and D_2O (as specified per spectrum).

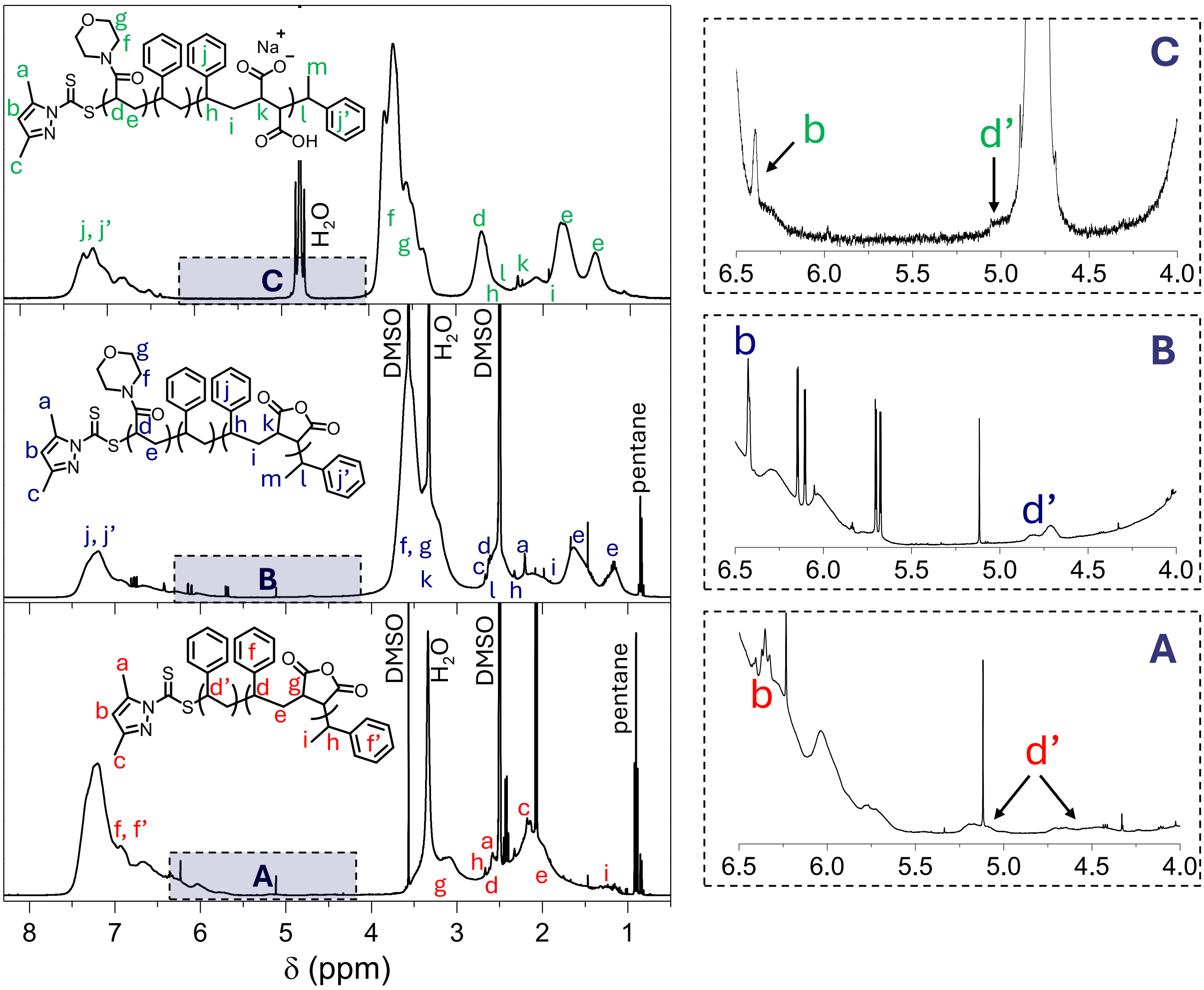

Figure S7. ¹H NMR spectroscopic analysis (600 MHz) of PSMAnh (bottom), PSMAnh-*b*-PNAM (middle) and PSMA-*b*-PNAM (top) after BCP hydrolysis, in DMSO-d_6 and D_2O (as specified per spectrum).

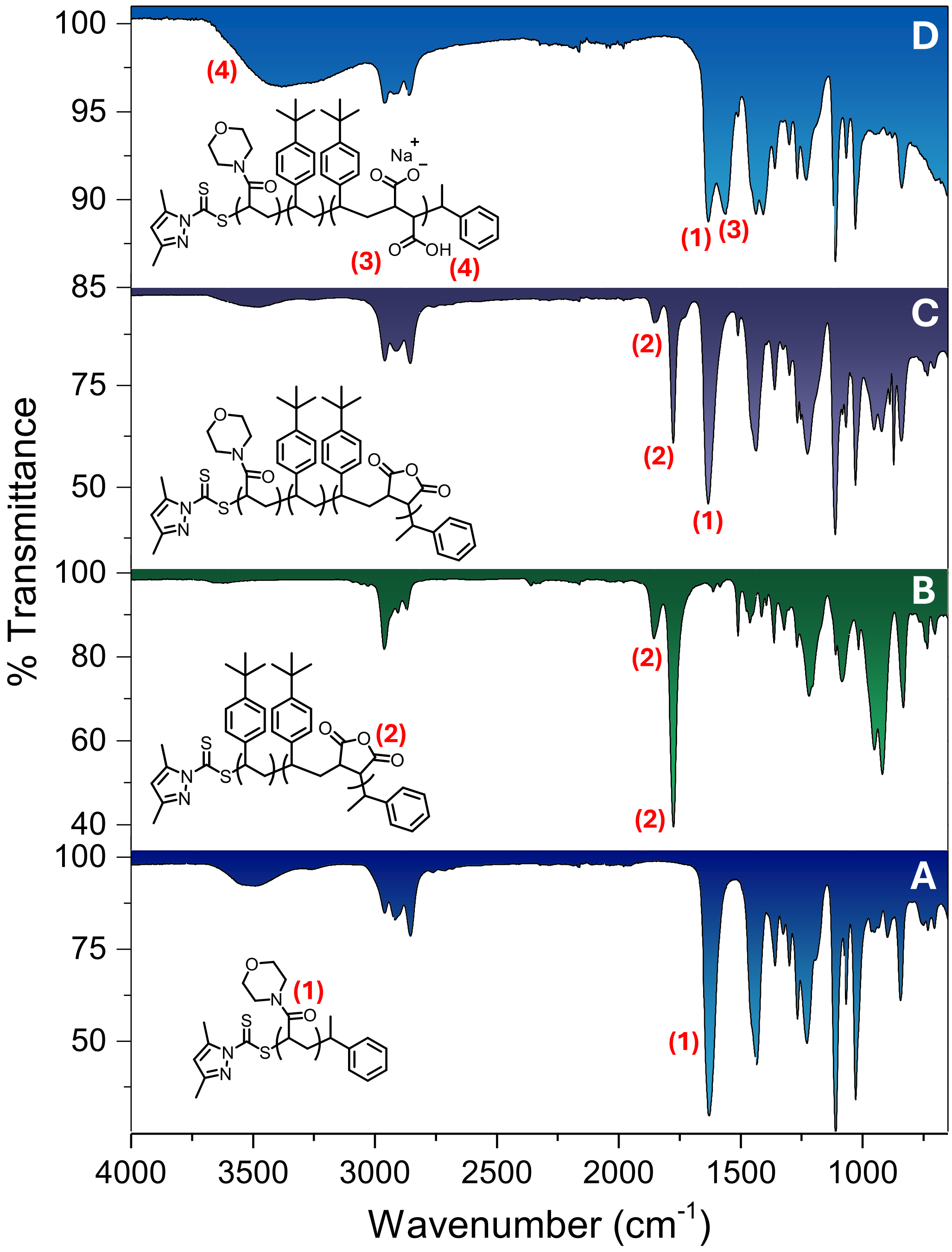

Figure S8. ATR-FTIR spectroscopic analysis of A) PNAM where (1) indicates the C=O stretching frequency (1630 cm^{-1}) of the amide carbonyl functional group, B) P*t*BuSMAnh where (2) indicates the C=O stretching frequencies (1856 and 1775 cm^{-1}) of the anhydride functional group, C) P*t*BuSMAnh-*b*-PNAM where both (1) and (2) are observed and D) P*t*BuSMA-*b*-PNAM after 10 min in the alkaline hydrolysis reaction mixture and 20 min drying under vacuum, indicating rapid and complete hydrolysis of MAnh units to MAc units, as (2) disappears and a new C=O stretching frequency (1564 cm^{-1}) (3) and broad O-H stretching frequency (3673–3050 cm^{-1}) (4) appear. It should also be noted that the C=O stretching frequency corresponding to the amide groups of the PNAM block in (D) shifts to a higher frequency (1633 cm^{-1}) due to the prevalence of hydrogen bonding between the PNAM and P*t*BuSMA blocks (as hydrogen bond acceptor and donor blocks, respectively).

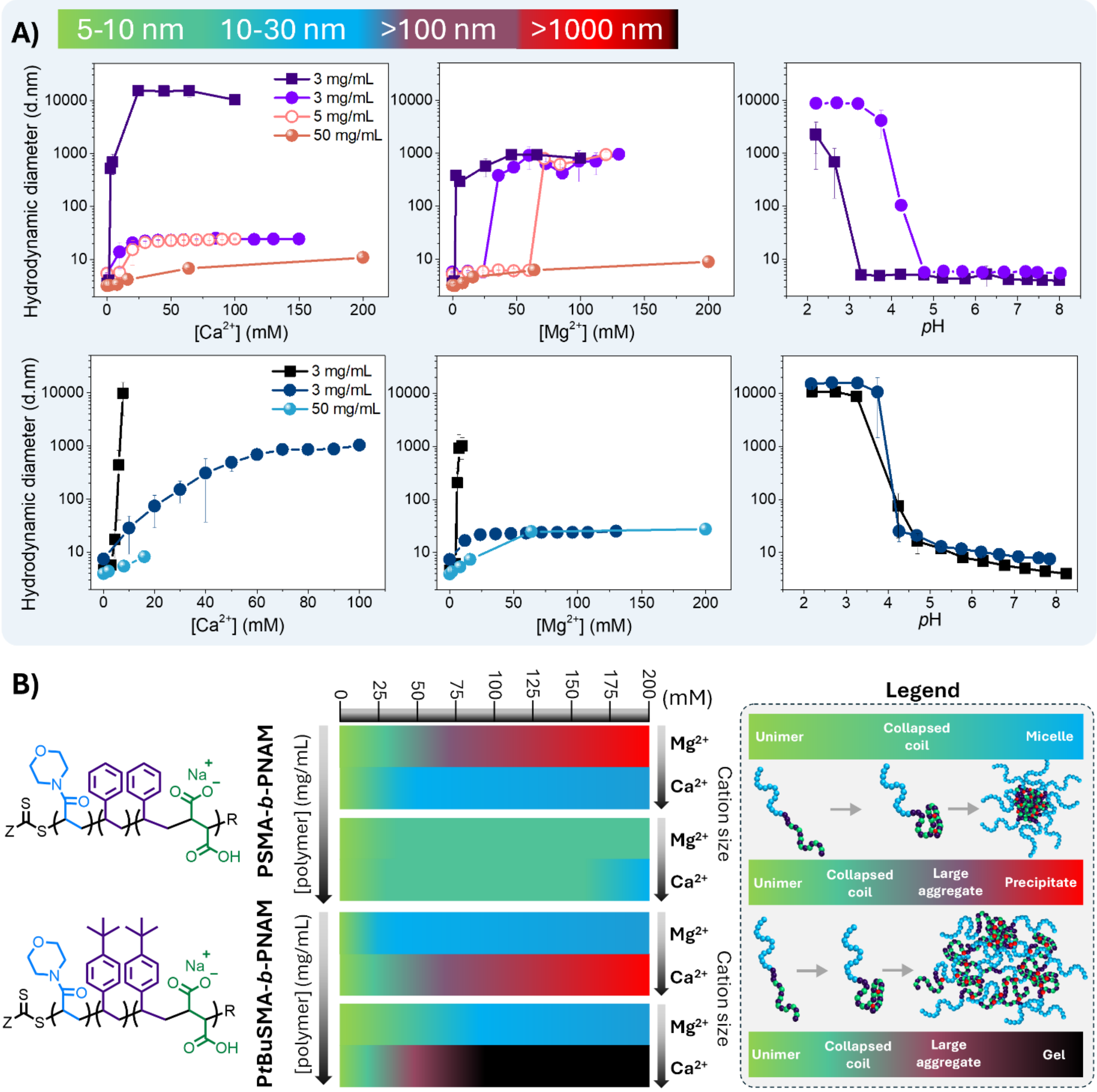

Figure S9. A) DLS analysis for PSMA (dark purple squares) and PSMA-*b*-PNAM (purple and pink circles) or P*t*BuSMA (black squares) and P*t*BuSMA-*b*-PNAM (blue circles) or at varying [M^{2+}] and polymer concentration. P*t*BuSMA-*b*-PNAM (blue circles) gels above 20 mM Ca^{2+} and therefore data points are not recorded up to 100 mM Ca^{2+}. B) Graphical summary of the aggregation behavior of the DHBCs with varying cation type, cation concentration and polymer concentration.

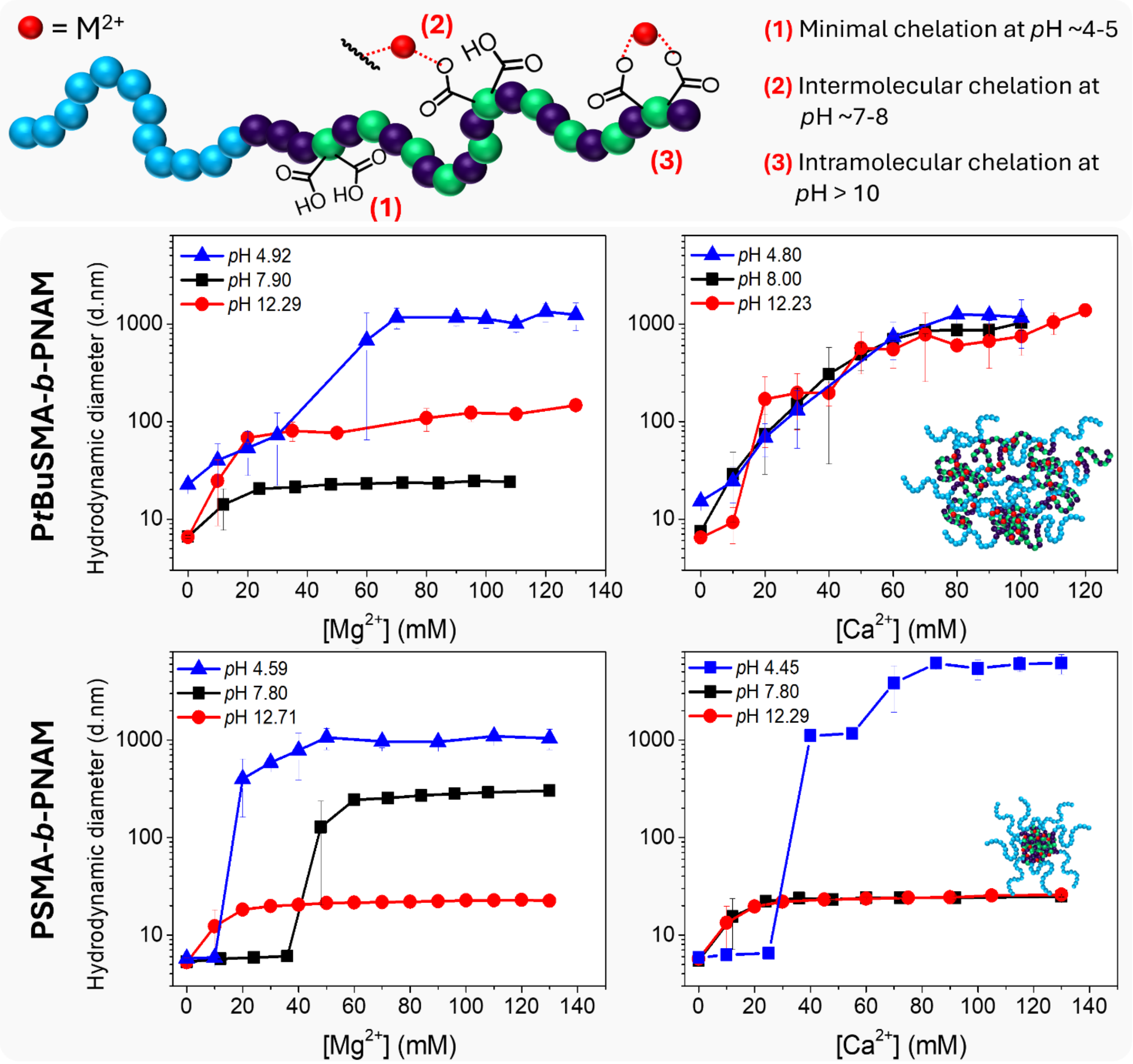

Figure S10. Titration of P*t*BuSMA-*b*-PNAM and PSMA-*b*-PNAM (at 3 mg/mL in DI water, 150 mM NaCl) with $CaCl_2$ (0.4 M) or $MgCl_2$ (0.4 M) at varying *p*H. The hydrodynamic diameter of the copolymers was assessed *via* DLS (fitted with an auto-titrator).

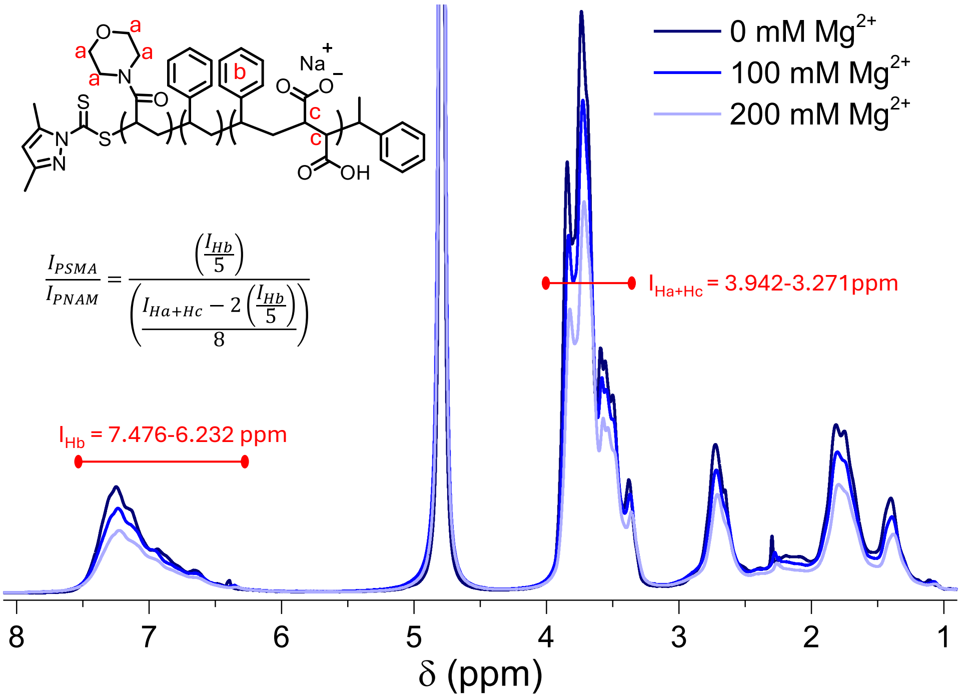

Figure S11. ^{1}H NMR spectroscopic analysis (600 MHz) of PSMA-*b*-PNAM at three different Mg^{2+} concentrations (0, 100 and 200 mM) in D_2O (150 mM NaCl). The shielding of PSMA protons due to micellization is determined *via* the ratio of PSMA and PNAM proton integrations, specifically aromatic STY protons (H_b) and the CH_2 protons of the PNAM morpholine ring (H_a), the latter of which overlaps with MAc protons (H_c).

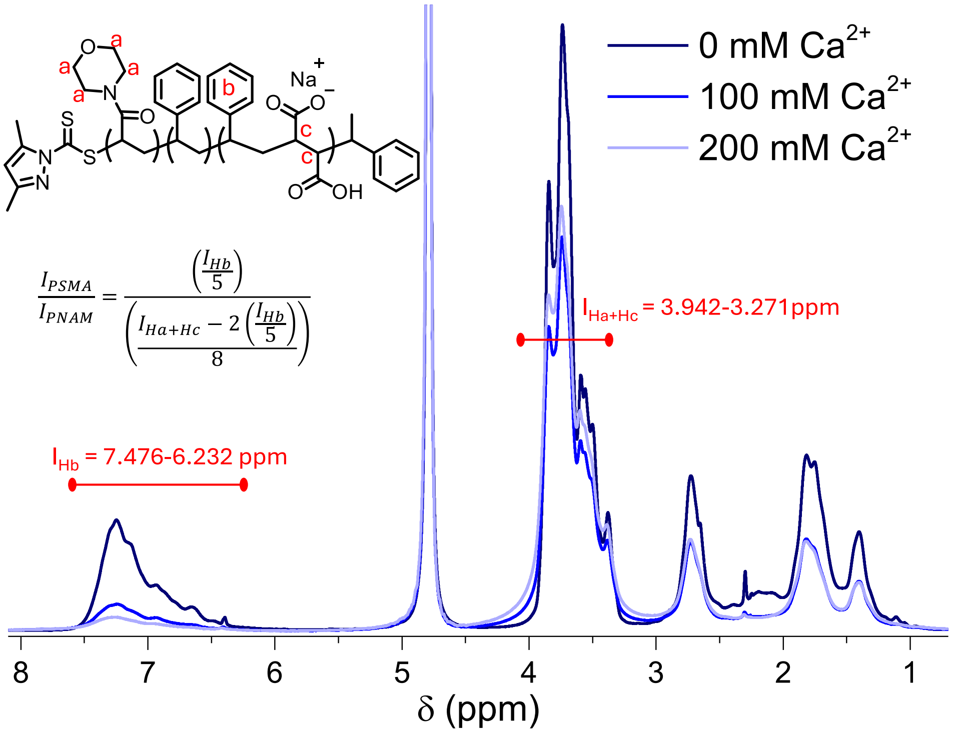

Figure S12. ^{1}H NMR spectroscopic analysis (600 MHz) of PSMA-*b*-PNAM at three different Ca^{2+} concentrations (0, 100 and 200 mM) in D_2O (150 mM NaCl).

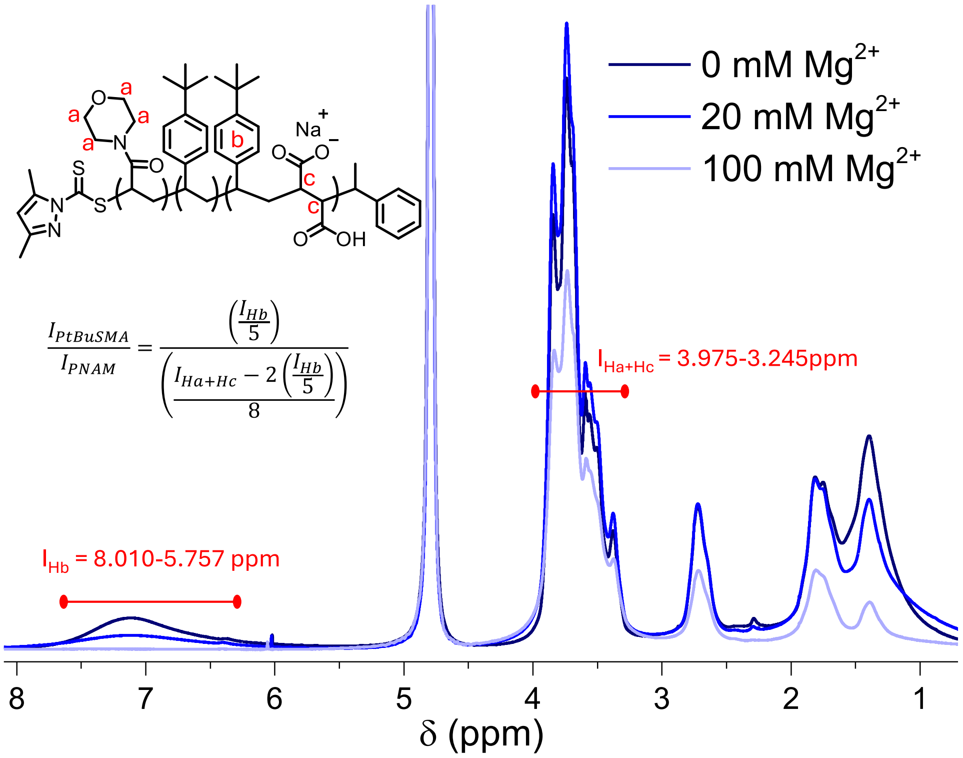

Figure S13. ^{1}H NMR spectroscopic analysis (600 MHz) of P*t*BuSMA-*b*-PNAM at three different Mg^{2+} concentrations (0, 20 and 100 mM) in D_2O (150 mM NaCl).

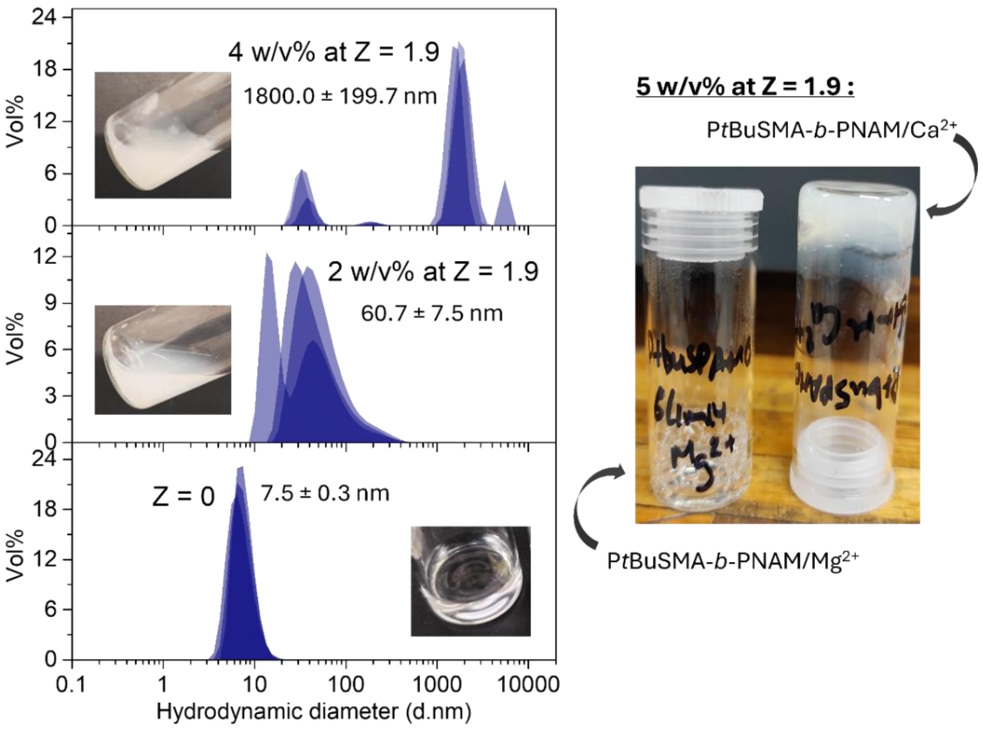

Figure S14. DLS analysis (left) for P*t*BuSMA-*b*-PNAM (Z = 0), P*t*BuSMA-*b*-PNAM/Ca^{2+} at 2 w/v% (Z = 1.9) and P*t*BuSMA-*b*-PNAM/Ca^{2+} at 4 w/v% (Z = 1.9). P*t*BuSMA-*b*-PNAM/Mg^{2+} and P*t*BuSMA-*b*-PNAM/Ca^{2+} at 5 w/v% (Z = 1.9) yield micelles and a hydrogel, respectively. The P*t*BuSMA-*b*-PNAM/Ca^{2+} hydrogel passes the vial-inversion test as the material resists flow for more than 1 min. The polymer observed on the sides of the vial were not the result of the material flowing, but due to the initial agitation of the mixture upon addition of the Ca^{2+} solution to the polymer solution.

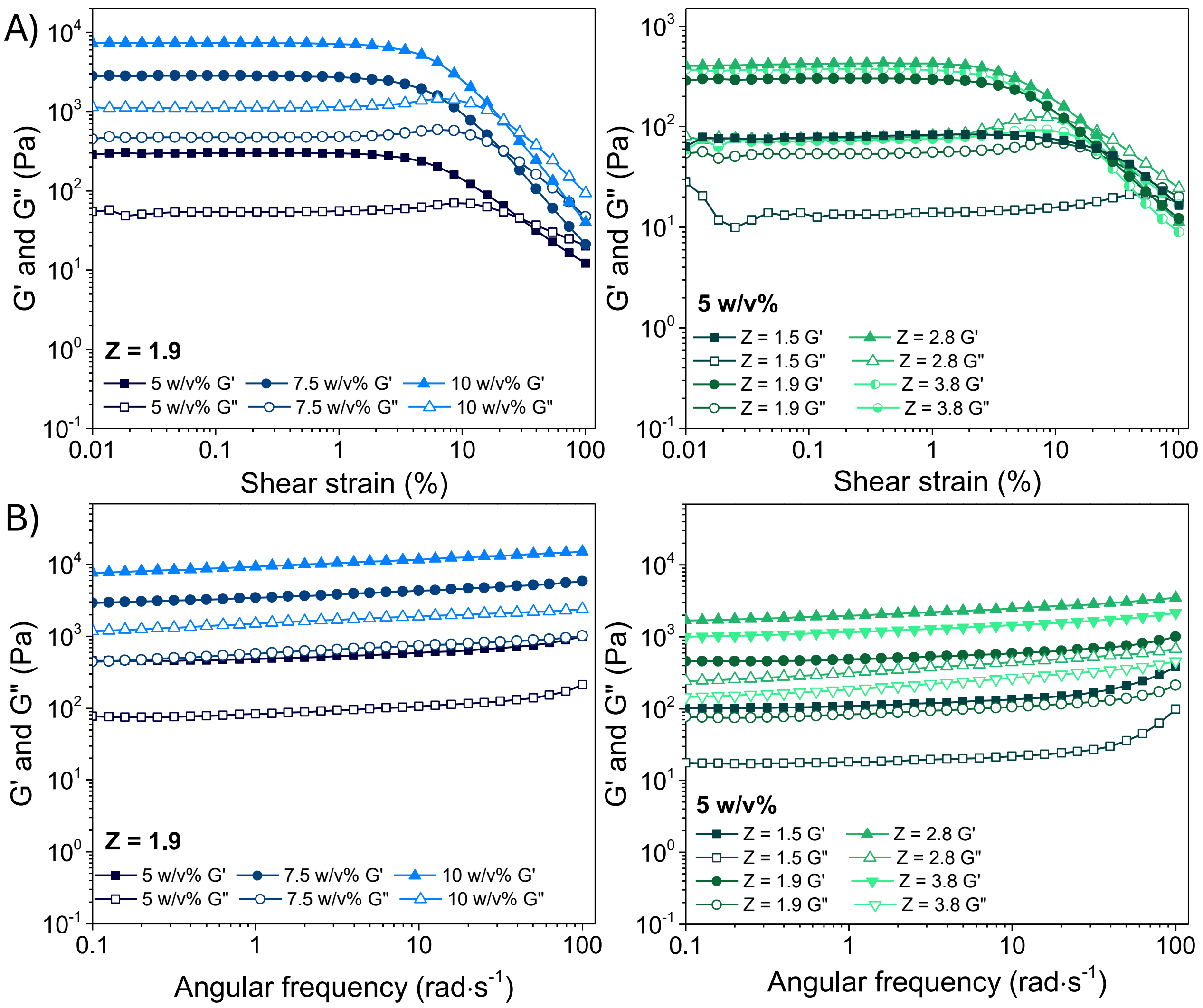

Figure S15. Rheological analyses for P*t*BuSMA-*b*-PNAM/Ca^{2+} gels. A) Strain sweep measurements for the determination of LVE region and B) for frequency sweeps at varying concentration of block copolymer (5–10 w/v%) while maintaining a constant Z and varying Z ([Ca^{2+}] = 52–128 mM) while maintaining a constant polymer concentration. All samples with varying polymer concentration at Z = 1.9 exhibited storage moduli independent of the strain amplitude up to ~4% after which then started yielding. Interestingly, P*t*BuSMA-*b*-PNAM/Ca^{2+} (5 w/v %) at the lowest Z (1.5) had storage moduli independent of the strain amplitude up to ~9% after which yielding occurred, whereas increasing Z resulted in hydrogels which yielded at lower shear strain (~4%).

Gelation of P*t*BuSMA-*b*-PNAM

Generally, the introduction of divalent cations to either DHBC aqueous solution at an appropriate Z ratio facilitated micelle formation, but application of [Ca^{2+}] > 52 mM to P*t*BuSMA-*b*-PNAM (>5 w/v%) resulted in a rapid increase in the viscosity of the DHBC solution with the resulting "gel-like" material passing a vial-inversion test (**Figure S14**). Similar experiments performed with PSMA-*b*-PNAM/Ca^{2+} or P*t*BuSMA-*b*-PNAM/Mg^{2+} did not yield a gel but rather facilitated the adoption of a collapsed coil conformation followed

by self-assembly into micelles, *vide supra*. It was hypothesized that the steric bulk of the *tert*-butyl groups promoted intermolecular chelation between MAc units and the larger Ca^{2+} cation for P*t*BuSMA-*b*-PNAM/Ca^{2+} complexes. It is plausible that the chelation and prevalence of hydrophobic domains act as physical crosslinks. These interactions, in combination with inter- and intramolecular hydrogen bonding between P*t*BuSMA and PNAM blocks, facilitated the effective gelation of P*t*BuSMA-*b*-PNAM/Ca^{2+} at relatively low polymer concentration and molecular weight. Owing to the observed "gel-like" behavior, these materials were probed using rheology. Amplitude sweeps, and subsequently frequency sweeps of P*t*BuSMA-*b*-PNAM/Ca^{2+} were carried out to assess the effect of polymer concentration and [Ca^{2+}] on the mechanical properties of the gels, the results are presented in **Figure S15**.

A critical gelation concentration of 5 w/v% P*t*BuSMA-*b*-PNAM and 52 mM Ca^{2+} (Z = 1.5) was determined, as the utilization of lower polymer content or smaller Z ratios resulted in the formation of a viscous free-flowing solution (**Figure S14**). Subsequently, the concentration of P*t*BuSMA-*b*-PNAM/Ca^{2+} was varied between 5–10 w/v% (while maintaining a constant Z ratio) or the Z ratio was varied between 1.5–3.8 (while maintaining a constant polymer concentration). For all samples assessed, the storage modulus (*G'*) was significantly greater than the loss modulus (*G"*), suggesting that the gels exhibit predominantly elastic behavior in the assessed frequency range. Incrementally higher values for *G'* and *G"* could be achieved by utilizing increasing concentrations of P*t*BuSMA-*b*-PNAM/Ca^{2+} (**Figure S15B**), however, *G'* and *G"* still showed a weak frequency dependence in the assessed frequency range (0.1–100 rad·s^{-1}). Increasing the Z ratio between 1.5–2.8 (52–96 mM Ca^{2+}) while maintaining P*t*BuSMA-*b*-PNAM at 5 w/v% also improved the mechanical properties of the gel as higher gel-stiffness was observed, but increasing the Z ratio further to 3.8 (128 mM Ca^{2+}) did not have a significant effect on the stiffness of the gel. The presented data suggest that the presence of the bulky hydrophobic *tert*-butyl group and a larger cationic radius are ideal for gelation, however, the exact mechanism of gelation is unclear. Therefore, it suffices to opine that chelation, hydrogen bonding and hydrophobic interactions collectively play a crucial role in the formation of the gels.

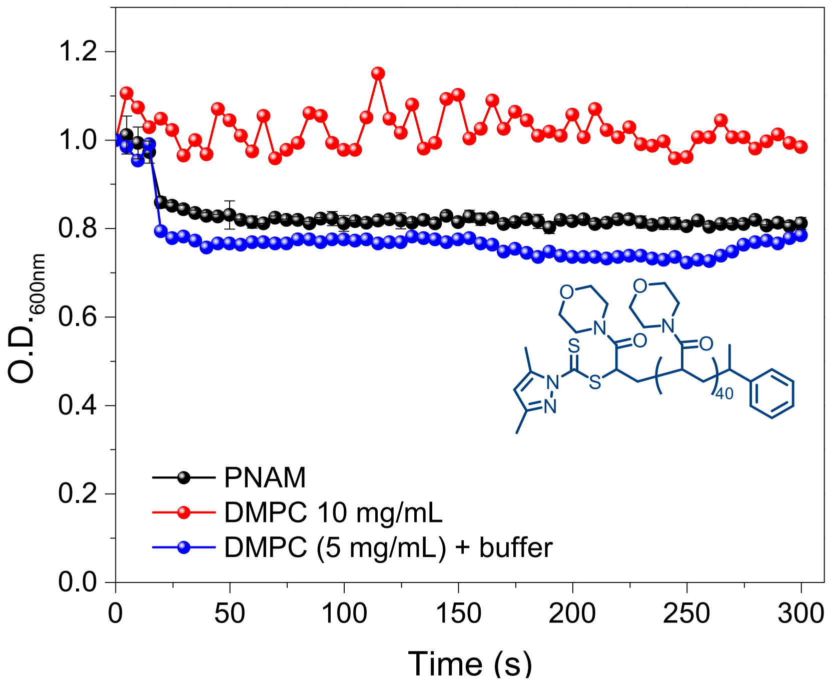

Figure S16. Turbidimetric analysis of DMPC vesicles (10 mg/mL in Tris HCl buffer, 50 mM, *p*H 7.6) treated with PNAM (50 mg/mL in Tris HCl buffer, 50 mM). A decrease in O.D.$_{600nm}$ is observed upon addition of PNAM to the vesicle solution, but purely as a result of sample dilution as a similar drop in O.D.$_{600nm}$ was observed for the DMPC solution treated with an equivalent volume of Tris HCl buffer.

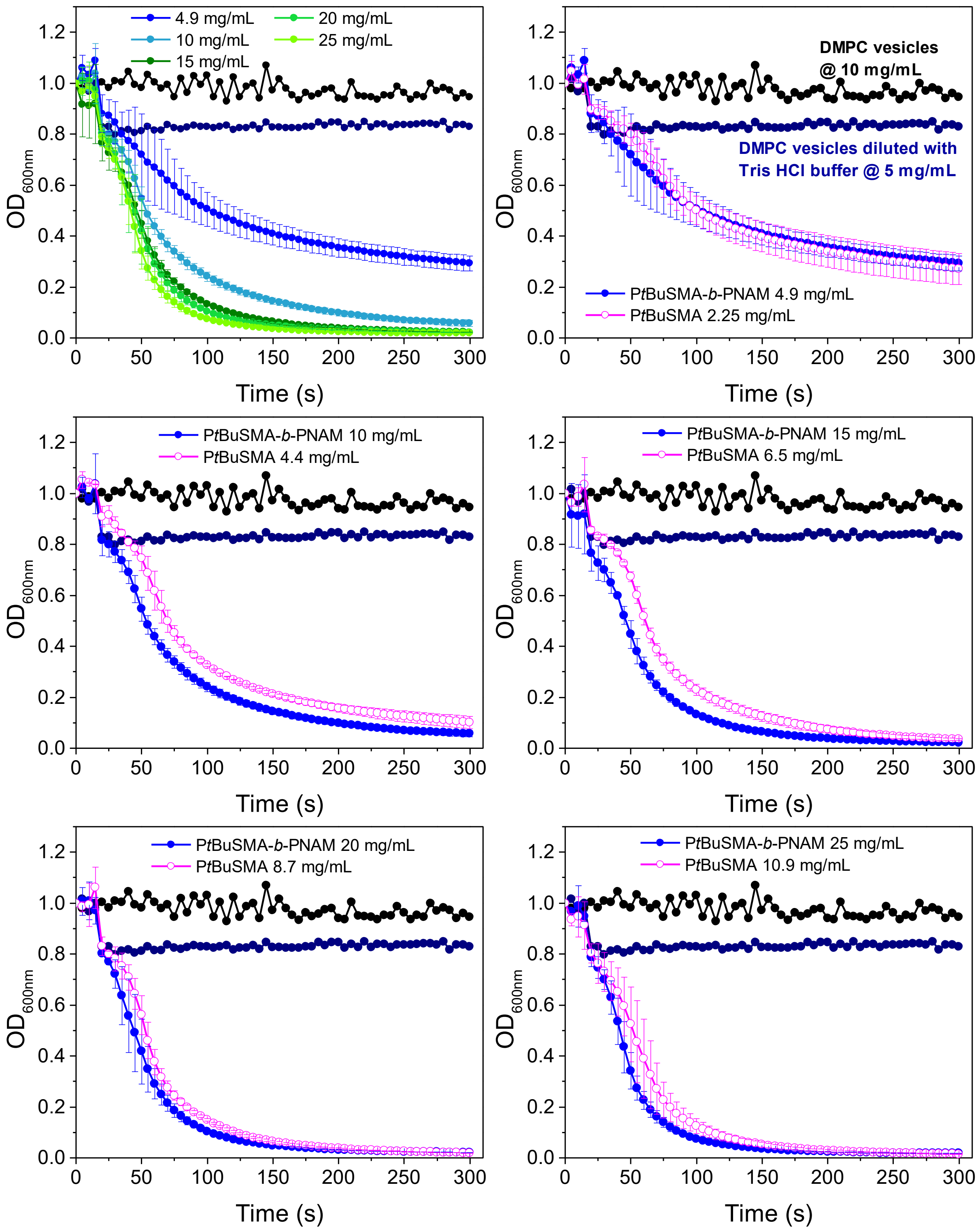

Figure S17. Turbidimetric analysis of the solubilization of DMPC vesicles (10 mg/mL in Tris HCl buffer, 50 mM) using P*t*BuSMA and its corresponding block copolymer P*t*BuSMA-*b*-PNAM. The normalized optical density is reported for a 5 min analysis (600 nm, 25 °C), with all samples analyzed in triplicate. The OD_{600nm} of the DMPC vesicles is monitored first, followed by the addition of an equivalent volume of Tris HCl buffer to show the effect of dilution on the optical density. Subsequent experiments assessed the optical density of DMPC vesicles for ~15 seconds followed by the addition of polymer at varying concentrations (in Tris HCl buffer, *p*H 7.9–8.1). The concentration of P*t*BuSMA-*b*-PNAM varied between 5-25 mg/mL (top left), where equivalent experiments were performed using P*t*BuSMA at a similar concentration to the P*t*BuSMA content

of the block copolymer (calculated via determination of the w/w% contribution of P*t*BuSMA in P*t*BuSMA-*b*-PNAM). Increasing the concentration of P*t*BuSMA-*b*-PNAM results in faster solubilization of the DMPC vesicles and comparable solubilization kinetics were observed for P*t*BuSMA and P*t*BuSMA-*b*-PNAM at any given concentration of polymer.

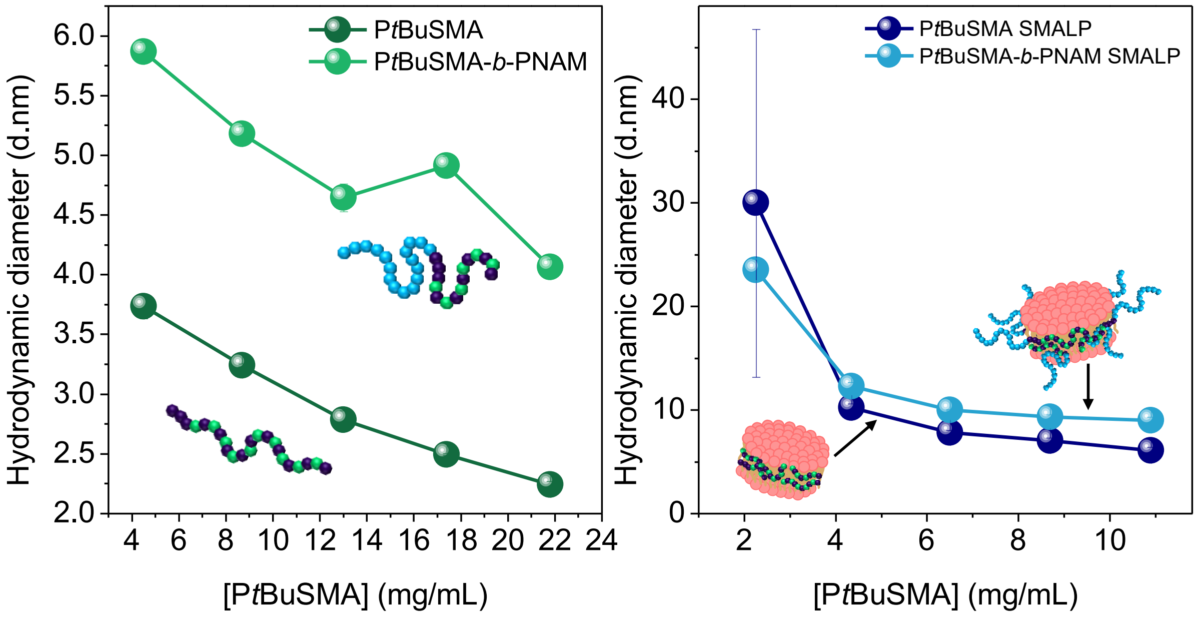

Figure S18. DLS analysis of P*t*BuSMA and P*t*BuSMA-*b*-PNAM copolymers (left) and SMALPs synthesized using P*t*BuSMA or P*t*BuSMA-*b*-PNAM (right). The concentration of polymer utilized has been reported as the concentration of P*t*BuSMA in the polymer (calculated according to the w/w% contribution of the P*t*BuSMA block in the block copolymer). The concentration of DMPC lipid is held constant at 10 mg/mL and after addition of the polymers (left), a final concentration of 5 mg/mL DMPC lipid is obtained (right). In most instances, the block copolymer results in SMALPs with slightly larger hydrodynamic diameter, ranging in size between 9–24 nm. The SMALP solution is then analyzed using DLS, *via* dilution of 10 µL aliquot of the sample in 1 mL Tris HCl buffer.

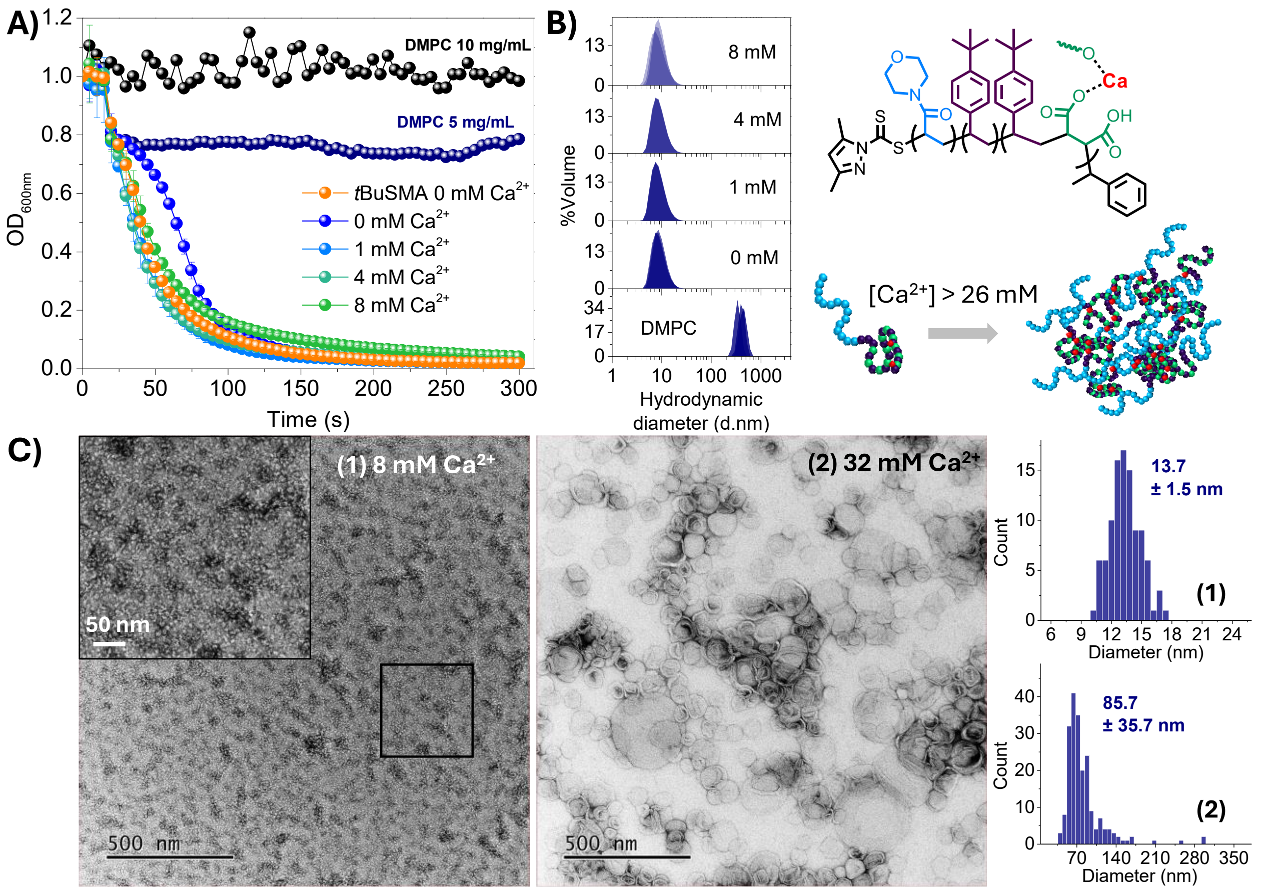

Figure S19. DMPC vesicles are prepared at 10 mg/mL (in Tris HCl buffer, 50 mM, *p*H 7.8) and polymer solutions (50 mg/mL) at varying [Ca^{2+}] (0–64 mM) are prepared separately. Once the two solutions are mixed a final DMPC and polymer concentration of 5 mg/mL and 25 mg/mL are obtained, respectively within a [Ca^{2+}] range of 0–32 mM. **A)** Turbidimetric analysis (600 nm at 25 °C, 5 min) for the solubilization of DMPC vesicles using P*t*BuSMA-*b*-PNAM at varying concentrations of Ca^{2+} (final concentrations between 0–8 mM). The polymer prepared at 64 mM Ca^{2+} (final concentration of 32 mM Ca^{2+}) forms a gel and therefore light scattering analyses could not be performed effectively. **B)** DLS analysis of P*t*BuSMA-*b*-PNAM SMALPs synthesized at [Ca^{2+}] = 0–8 mM, indicating a decrease in hydrodynamic diameter from 403 ± 53 nm (DMPC vesicles) to ~10 nm (SMALPs). **C)** TEM micrographs of DMPC and polymer mixtures at [Ca^{2+}] = 8/32 mM. The sample at 32 mM Ca^{2+} was prepared by mixing the gel-like polymer sample (at 64 mM Ca^{2+}) with DMPC vesicles (10 mg/mL), where the resulting TEM micrograph indicates the presence of deflated vesicular structures.

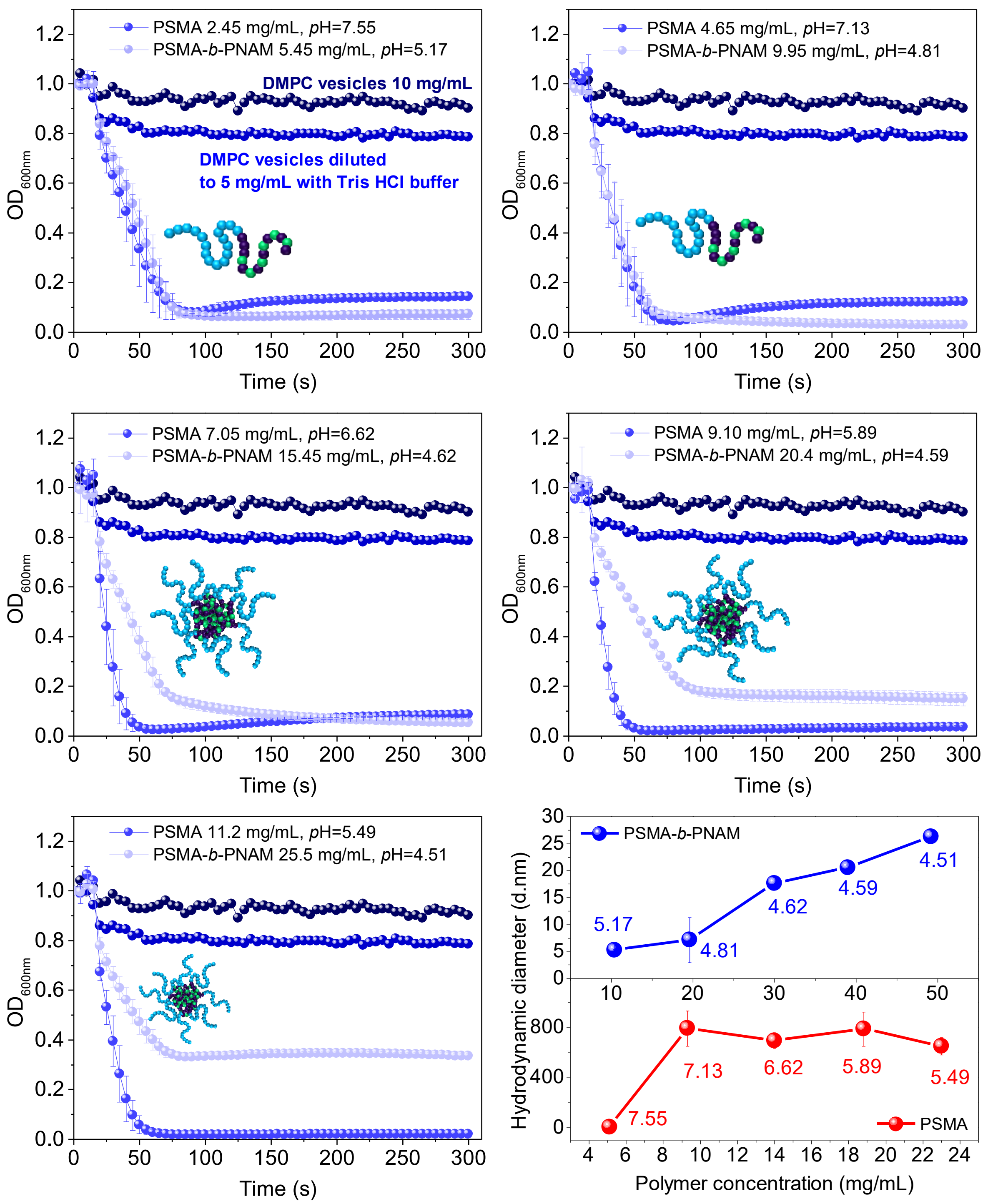

Figure S20. Turbidimetric analysis of the solubilization of DMPC vesicles (10 mg/mL in Tris HCl buffer, 50 mM) using PSMA and its corresponding block copolymer PSMA-*b*-PNAM. The normalized optical density is reported for a 5 min analysis (600 nm, 25 °C), with all samples analyzed in triplicate. The OD_{600nm} of the DMPC vesicles is monitored first, followed by the addition of an equivalent volume of Tris HCl buffer to show the effect of dilution on the optical density. Subsequent experiments assessed the optical density of DMPC vesicles for ~15 seconds followed by the addition of polymer at varying concentrations (in Tris HCl buffer, *p*H 4.5–5.2). The concentration of PSMA-*b*-PNAM varied between 5-25 mg/mL, where equivalent experiments were performed using PSMA at a similar concentration to the PSMA content of the block copolymer (calculated *via* determination of the w/w% contribution of PSMA in PSMA-*b*-PNAM). The

alternating PSMA copolymer is generally too hydrophilic for the effective solubilization of DMPC vesicles, so the dose response analysis was conducted at acidic *p*H to increase the hydrophobicity of the PSMA block. This facilitated micelle formation at block copolymer concentrations above 30 mg/mL (18–26 nm), which corresponds to a final polymer concentration of 15 mg/mL and above for the solubilization experiments. The application of acidic *p*H caused the aggregation of PSMA into stable latexes (~800 nm) in solution. The solubilization kinetics of PSMA and PSMA-*b*-PNAM were comparable when unimeric block copolymer chains were present, but significantly slower solubilization kinetics were observed when the block copolymer was present as a micelle. An apparent difference in stability of the structures formed was also observed, as the optical density dropped to near zero for PSMA samples (at lower concentrations) followed by a gradual increase in OD_{600nm}. This was not observed for PSMA-*b*-PNAM samples, which exhibited a drop in OD_{600nm} near zero, with no subsequent increase in optical density throughout the 5 min analysis.

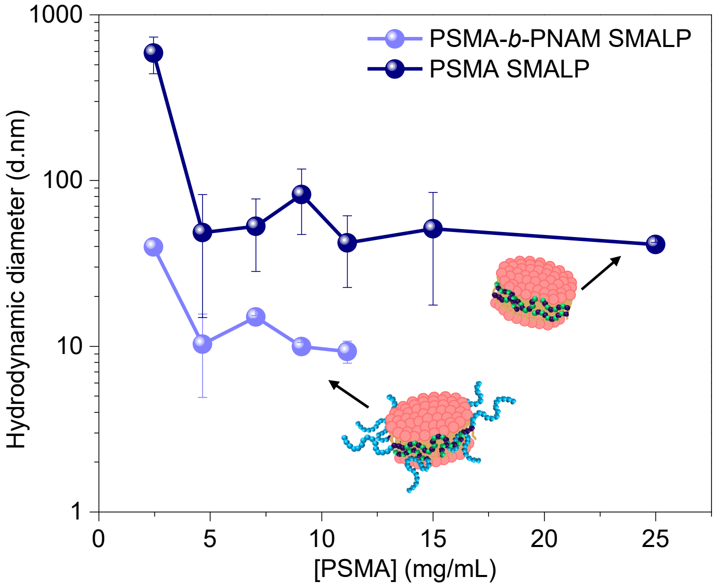

Figure S21. DLS analysis of SMALPs synthesized using PSMA or PSMA-*b*-PNAM (solubilizations in **Figure S20**). The concentration of polymer utilized has been reported as the concentration of PSMA in the polymer (calculated according to the w/w% contribution of the PSMA block in the block copolymer). The concentration of DMPC lipid is held constant at 10 mg/mL and after addition of the polymers, a final concentration of 5 mg/mL DMPC lipid is obtained.

References

1. Ball, L. E., Smith, M. P., Pfukwa, R. & Klumperman, B. An Exploration of the Universal and Switchable RAFT-Mediated Synthesis of Poly(styrene-alt-maleic acid)-b-poly(N-vinylpyrrolidone) Block Copolymers. *Macromolecules* 58, 1060–1078 (2025).
